# Characterizing the luminosity components of luminous infrared galaxies in multi-wavelength from the X-ray to the far-infrared

**DOI:** 10.1038/s41598-024-76203-5

**Published:** 2024-10-27

**Authors:** Abdallah A. M. Ali, Kamel A. K. Gadallah, Osama. M. Shalabiea, Mohamed. M. Beheary

**Affiliations:** 1 Astronomy and Meteorology Department, Faculty of Science, Al-Azhar University, PO Box 11884, Nasr City, Cairo, Egypt; 2 Astronomy, Space Science and Meteorology Department, Faculty of Science, Cairo University, Giza, Egypt; 3 Faculty of Navigation Science and Space Technology, Beni-Suef University, Beni-Suef, Egypt

**Keywords:** Luminous IR galaxies, Active Galactic Nucleus (AGN), Stellar luminosity, X-ray luminosity, AGN Luminosity, Galaxies and clusters, High-energy astrophysics, Interstellar medium

## Abstract

We characterize luminosity components of Ultra/Luminous Infrared Galaxies (U/LIRGs) in multi-wavelength from the X-ray to far-infrared. A set of 63 AGN U/LIRGs was selected where these galaxies are powered by a central active galactic nucleus (AGN). Utilizing the X-CIGALE code, SEDs for these galaxies are carried out where their SEDs are fitted with observations. Accordingly, the physical parameters such as the stellar mass, the dust-to-gas mass ratio, and the star formation rate are calculated. The total luminosity and its decomposed components (stellar, AGN, X-ray) are also calculated. We characterized these luminosities in relative to the intrinsic luminosity and in relative to each other. As a function of the stellar mass, these luminosities reveal an increase with different correlation coefficients, showing a strong correlation. In correlation with the intrinsic AGN power, the stellar, AGN, and X-ray luminosities are strongly correlates in their variation to the intrinsic AGN luminosity, showing stronger correlations of AGN, and X-ray luminosities than those of the stellar one. In relationships between various luminosity components, both the stellar and X-ray luminosities reveal strong correlations with the AGN luminosity. On the other hand, the X-ray luminosity varies strongly with the stellar luminosity and moderately with IR luminosity. Compared to obscured AGN galaxies, both the stellar and AGN luminosities similarly vary with increasing the intrinsic power of the active nucleus but for obscured AGN they are faster in their variation than that of U/LIRG. These correlations may offer valuable insights to understand the physical properties and their relationships through the evolution of U/LIRGs.

**Supplementary Information:**

The online version contains supplementary material available at 10.1038/s41598-024-76203-5.

## Introduction

LIRGs and ULIRGs are a unique class of galaxies that exhibit extremely high infrared luminosities (LIR) of > 10^11^*L*_☉_ for LIRG and > 10^12^*L*☉ for ULIRG^1–3^. These galaxies are often associated with intense star formation and the existence of Active Galactic Nucleus (AGN), which significantly represent their energy sources. AGN multiwavelength properties, as summarized in overviews of^4^, are induced from various physical processes. Observations, utilizing the James Webb Space Telescope (JWST) and Near Infrared Spectrograph (NIRSpec), provide an evidence of high ionization rates in U/LIRGs, indicating a more complex interplay between star formation and AGN activity^5^. Understanding the characteristics and the physical properties when they host AGN is crucial for unraveling the complex interplay between star formation and AGN activity in these systems. U/LIRGs serve as valuable laboratories for investigating intense star formation and AGN activity that is heavily veiled by dust. These galaxies provide a unique opportunity to understand and interpret observations from analogous occurrences in the most luminous galaxies such as hyper luminous infrared galaxies^6,7^, submillimeter galaxies^7–9^and quasars^10–13^. Due to these various galaxies, U/LIRGs have garnered significant attention ever since their detection by the Infrared Astronomical Satellite (IRAS) in the 1980s^14,15^.

AGN activity in LIRGs or ULIRGs plays a pivotal role in shaping their Spectral Energy Distributions (SEDs). The unified model of AGN^16–18^ suggests that AGNs consist of a central supermassive black hole surrounded by an accretion disk and a torus of obscuring dust and gas. The interaction between the AGN and the surrounding environment, including the torus, influences the observed SED properties of LIRGs and ULIRGs. Observations further enhance our understanding of outflows and AGN activities in ULIRGs providing new insights into AGN-driven feedback mechanisms^19^.

SED analysis provides a comprehensive understanding of the energy output and physical properties of galaxies across a wide range of wavelengths^20^. In recent years, the investigation of SEDs has played a crucial role in studying U/LIRGs hosting AGN (e.g^21^). These galaxies exhibit intense infrared emission, indicating the presence of powerful AGN and/or vigorous star formation activities^22^. The analysis of radio spectral characteristics, as explored in^23^, provides additional insights into the star formation history and AGN activity within ULIRGs, further refining their classification.

The AGN of a galaxy having a dusty torus plays a crucial role in the AGN emissions from certain angles, particularly in the X-ray/UV/optical^16^ and in ranges from X-ray to Far Infrared range (e.g^24^). The absorbed energy by the torus is re-emitted as thermal radiation in the infrared (IR) band, leading to the characteristic IR emission observed in AGNs^25^. The SED spectra of U/LIRGs are strongly characterized by their MIR emissions due to the existence of dust. This dust mainly consists of small grains of polycyclic aromatic hydrocarbons (PAHs) and large grains of silicate and carbonaceous materials^26–29^ where the UV radiation heats these grains^30,31^.

Numerous efforts have been made to elucidate the relationship between the AGN and their host galaxies, particularly in the context of X-ray background radiation^32^ and on the degree of obscuration linked to the geometric structure of the dusty torus surrounding the AGN^33^. Several recent investigations^34–36^ have highlighted the correlation between X-ray and mid-infrared (MIR) emissions.

The SED curves of AGNs are shaped by a power-law distribution across multiple wavelengths, encompassing absorptions and emissions from various sources within the galaxy^37^. The UV/optical/NIR ranges are primarily dominated by stellar emissions, offering insights into the star-formation history and attenuation caused by dust. AGNs exhibit a distinctive X-ray emission which is widely utilized as a tracer of the black hole accretion rate^38^. The FUV-FIR range in the galaxy SED contains nebular lines and continuum emissions, arising from Lyman continuum photons, particularly by hydrogen lines, and is vital in tracing recent star formation^39,40^. The MIR range contributes fractionally to AGN emission where the MIR-FIR range presents dust processing due to absorption/emission of starlight. In this study, a combination of toroidal and polar dust with smooth and clumpy phases is assumed to reproduce SEDs in a broad range of emissions from X-ray to Far-Infrared (FIR). In multi-wavelengths from hard X-Ray to Radio, the SED physical properties of U/LIRGs have been comprehensively studied by^41^ considering their merger evolution, where their radio band is dominated by the starburst emission. For the radio-to-far-ultraviolet SEDs of ULIRGs, recent study by^23^ provides new insights into the global properties of infrared-bright galaxies.

For a model of a U/LIRG radiatively fueled by an active nucleus and surrounded by a dusty torus, we are motivated to describe the variation of the SED luminosity components with the intrinsic AGN luminosity and the stellar mass of the host galaxy. For which, decomposing the SED spectrum is considered where the various emissions are released across the X-ray to the FIR. Both SED model and the galaxy sample are given in Sects. 2 and 3, respectively. In Sects. 4 and 5, we present the results and discussion of the SED model outputs, respectively, while the conclusion is summarized in Sect. 6.

## The SED model

To generate SED spectra of LIRG/ULIRG galaxies in ranges from X-ray to FIR band, the PYTHON Code Investigating GALaxy Emission (CIGALE) developed by^42^ was used, considering the modification of the last version (X-CIGALE) by^43,44^. This version incorporated the X-ray band by adding an X-ray photometry module and a module for the polar dust. This code can calculate the galaxy physical properties such as stellar, gas, and dust masses, star formation rate, as well as the luminosity components of stellar, AGN, and X-ray emissions.

To represent the contributions of the galactic disc components to the galaxy SED, several modules were selected, covering aspects such as star-formation history, stellar populations, nebular emission, attenuation law, dust emission, and active nucleus. Detailed descriptions of these modules and their physical parameters based on the assumptions of^42,43]^ and^44^, are given in (e.g^24^).

For LIRG/ULIRG galaxies, the parameters of selected modules were adapted to fit them with observational data from the X-ray to the FIR range. To produce their SED, a module (*sfhdelayedbq*) of delayed star formation history was used for flexibility by considering recent quenching of the star formation rate (SFR). This module enables the SFR to increase from the start of time (*t*) reaching a peak at *t* = *τ*_main_, which represents the e-folding times of the stellar populations. The SFR was estimated following the assumptions of^45^,

1 $$\:\text{S}\text{F}\text{R}\left(t\right)\propto\:\left\{\begin{array}{c}{te}^{-t/{\tau\:}_{main}},\:\:\:\:\:\:\:\:\:\:\:\:\:\:\:\:\:\:\:\:\:\:\:\:\:if\:\:\:t\le\:0\\\:{r}_{SFR}\:\times\:\:SFR\left(t={t}_{0}\right),\:\:\:\:\:if\:\:t>0\end{array}\right.$$

where, $\:{t}_{0}$ represents the time at a significant increase or decrease in SFH, and $\:{r}_{SFR}$ denotes the ratio of the $\:\text{S}\text{F}\text{R}$ at $\:{t=t}_{0}$ to that at $\:t\:>\:0$.

To generate the intrinsic stellar spectrum for young and old stars with various metallicities and initial mass functions, the *bc03* module utilizes the stellar populations of^46^. Besides calculating stellar luminosity (*L*_st_) and mass, this module also estimates the gas mass (*M*_g_) parameter^47,48^. The gas mass specifically pertains to material ejected from stars, including stellar winds and supernovae, signifying the total gas mass returned to the interstellar medium through stellar evolution. Multicomponent photoionization models matching the emission lines can be generated by the *nebular* module relying on the adopted cloudy model^49,50^. The impact of attenuation laws in galaxies can be modeled using the *dustatt_modified_starburst* module proposed by^51^ which is better suited for applying the starburst curve. This introduces a power-law slope to modify the attenuation, along with a UV bump at 217.5 nm^20,52^. The *dl2014* module computes of the dust emission contribution to the SEDs based on the model proposed by^30,53^. Dust grains are assumed to be a combination of amorphous silicate, graphite, and PAHs where they are heated primarily from stellar populations by a minimum single radiation field (*U*_*min*_) in diffuse regions to its maximum value (*U*_*max*_) star-forming regions. The dust emission curve is defined through the utilization of a power-law index (*α*) and the dust mass-fraction (*γ*) when the dust is exposed to the maximum radiation *U*_*max*_. For the torus containing smooth and clumpy phases, the *skirtor2016* module^54^ is used to fit the SEDs of AGN emissions, considering approximately 97% of the dust mass (*M*_d_) and the remaining of the smooth phase^43^. adapted this module considering both the old and new essential luminosities, normalized to unity. The parametrization of this model is based on the physical and geometrical properties of the galactic disc, reproducing the galaxy SED for various lines of sight, and viewing angles. For which, type 2 AGN (edge-on galaxy) is obscured by the dusty torus along the equatorial direction whereas type 1 AGN (face-on galaxy) is directly visible but slightly obscured by polar dust clumps. To estimate the luminosity as a function of wavelength in the FUV to FIR range^43^, updated the SKIRTOR model using luminosities supported by observations^55^:

2 $$\:\lambda\:{L}_{\lambda\:}\propto\:\left\{\:\begin{array}{c}{\lambda\:}^{2},\:\:\:\:\:\:\:\:\:\:\:\:\:if\:\:\:0.008\le\:\lambda\:<0.05\:\mu\:m,\\\:{\lambda\:}^{0.8},\:\:\:\:\:\:\:\:\:\:\:if\:\:\:\:0.05\le\:\lambda\:<0.125\:\mu\:m,\:\\\:{\lambda\:}^{-0.5},\:\:\:\:\:\:\:\:\:\:if\:\:\:\:\:0.125\le\:\lambda\:<10\:\mu\:m\:,\\\:{\lambda\:}^{-3},\:\:\:\:\:\:\:\:\:\:\:if\:\:\:\:\lambda\:<10\:\mu\:m\end{array}\right.$$

A new *X-ray* module incorporates the X-ray spectrum of the AGN as well as of the galaxy components. It is designed to fit the X-ray data covering the range of 10^−6^ to 5 × 10^−3^ μm in the rest frame. The intrinsic AGN X-ray spectrum’s flux (*f*_*ν*_) is computed using a power-law form with a high-energy exponential cut-off as follows:

3 $$\:{f}_{\upsilon\:}\propto\:{E}^{-\varGamma\:+1}\text{e}\text{x}\text{p}\left(-E/{E}_{cut}\right)$$

where $\:\varGamma\:$, $\:E$ and $\:{E}_{cut}$are the photon index, the energy, and the cut-off energy, respecti vely. Other three sources contribute to X-ray emissions: low-mass X-ray binaries (LMXBs), high-mass X-ray binaries (HMXBs), and hot gas. In Eq. (3), X-ray emission arises from the AGN, LMXBs/HMXBs, and hot gas at cut-off energies ($\:{E}_{cut}$) of 300 keV, 100 keV, and 1 keV, respectively. Moreover, the estimates of the luminosities are considered to be viewing-angle-dependent where UV/optical emissions are assumed to be anisotropic and X-ray emissions to be isotropic. The photon index $\:{\Gamma\:}$ was taken as 1.8 for X-ray AGN emission. To represent the fitting between X-ray and other wavelength ranges, the deviation of the maximum deviation ($\:{\left|{{\Delta\:}}_{ox}\right|}_{max}$) was used based on the *α*_*ox*_ *− L*_*2500Å*_ relationship^56^. Here, *L*_*2500Å*_ refers to the de-reddened AGN luminosity at 2500 Å, and *α*_*ox*_ represents the SED slope between the UV at 2500 Å and X-ray at 2 keV.

The free parameters of the modules described above are summarized in Table 1 to build up the X-CIGALE code. This code performs these modules simultaneously to fit the SED spectrum. It estimates the galaxy’s physical parameters such as the luminosity components, the stellar mass of the host galaxy and dust-to-gas mass ratio by the SED models.

**Table 1:** The free parameters of the modules that build up the X-CIGALE code.

sfhdelayedbq module	
e-folding time τ(Gyr) Stellar age *t* (Gyr) Age of the burst/quench *t*_bq (Gyr) Ratio of the SFR after/before quenching (RSFR)	0.25, 0.5, 1, 2, 4, 6, 8, 10, 12 0.5, 1, 2, 3, 4, 5, 6, 7, 8, 9, 10, 11, 12, 13 100, 200, 300 0.25, 0.3, 1, 10, 20, 30, 70
***bc03*** module	
Initial mass function (imf) Metallicity	0^57^ or 1^58^ 0.0001, 0.004, 0.008, 0.02
***nebular*** module	
Ionization parameter (log *U*) Line width (km s^−1^)	−3, − 2, −1 100, 200, 300
***dustatt_modified_starburst*** module	
*E*(*B* − *V*) of starlight for young/old populations (mag) *E*(*B* − *V* ) factor of the stellar continuum attenuation Centre, width at FWHM of the UV bump Slope of the power law modifying the attenuation curve Extinction law (*R*_V_)	Adapted to be > 0 and < 1 Adapted to be > 0 and < 1 217.5 nm, 35 nm Adapted to be between − 0.5 and 0.5 1, 3.1
***dl2014*** module	
Mass fraction of PAH (*q*_PAH_) Minimum radiation field (*U*_*min*_) (Habing) αof the power-law slope (*dU/dM*$\:\propto\:$*U*^*α*^) Fraction illuminated from *U*_min_ to *U*_max_ (γ)	0.47, 1.77, 2.50, 3.19, 3.90, 6.63, 7.32 From 0.1 to 50 Adapted to be between 1.5 and 3 Adapted to be between 0 and 1
***skirtor2016*** module	
Torus optical depth at 9.7 μm, *τ*_9.7_ Power-law exponent that sets radial gradient of dust density Index that sets dust density gradient with polar angle Opening angle of the torus Viewing angle of the galaxy Ratio of outer to inner radius (*R*_out_ / *R*_in_) Fraction of total dust mass inside clumps AGN fraction in total IR luminosity Extinction law of polar dust *E*(*B* –*V*) for extinction in polar direction Temperature, emissivity index of the polar dust (K)	3, 5, 7 0., 1. 0., 1. 20^◦^, 40^◦^, 60^◦^ 0^◦^, 30^◦^ for type 1; 70^◦^, 90^◦^for type 2 10, 20, 30 97% Adapted to be between 0.001 and 0.8 1 ^51^ 0, 0.1, 0.2, 0.3, 0.4 100, 1.6
***X-ray*** module	
Photon index of AGN intrinsic X-ray spectrum (*Γ*) Max. deviation ($\:{\left|{{\Delta\:}}_{ox}\right|}_{max}$) from *α*_ox_ − *L*_2500Å_ relationship LMXB, HMXB photon index of low-, high-mass X-ray binaries	1.8 0.2, 0.4 1.56, 2.0

## Sample of galaxies

In Table A1 (Appendix A), listed a sample of 63 LIRG/UlIRG galaxies selected to perform SEDs for the current work. This sample includes a group of 53 galaxies taken from the Great Observatories All-sky LIRG Survey (GOALS^59^) which totally contains 180 LIRGs and 22 ULIRGs located in the local universe with redshifts *z* < 0.088. These galaxies are part of the IRAS Revised Bright Galaxy Sample (RBGS^60^), which is an extensive collection of 629 extragalactic objects showing 60 μm fluxes above 5.24 Jy at Galactic latitudes |b| > 5. These U/LIRGs have been extensively studied across multiple wavelengths, particularly, infrared observations carried out with the IR telescopes of Spitzer^61–63^, AKARI^64,65^, and Herschel^66–68^. Additionally, X-ray observations have been performed using the Chandra X-ray telescope^69,70^ and the NuSTAR X-ray telescope^71,72^. Another group consisting of 10 sources, with redshift *z* > 0.088, was collected from the literature^73–77^.

**Fig. 1:**
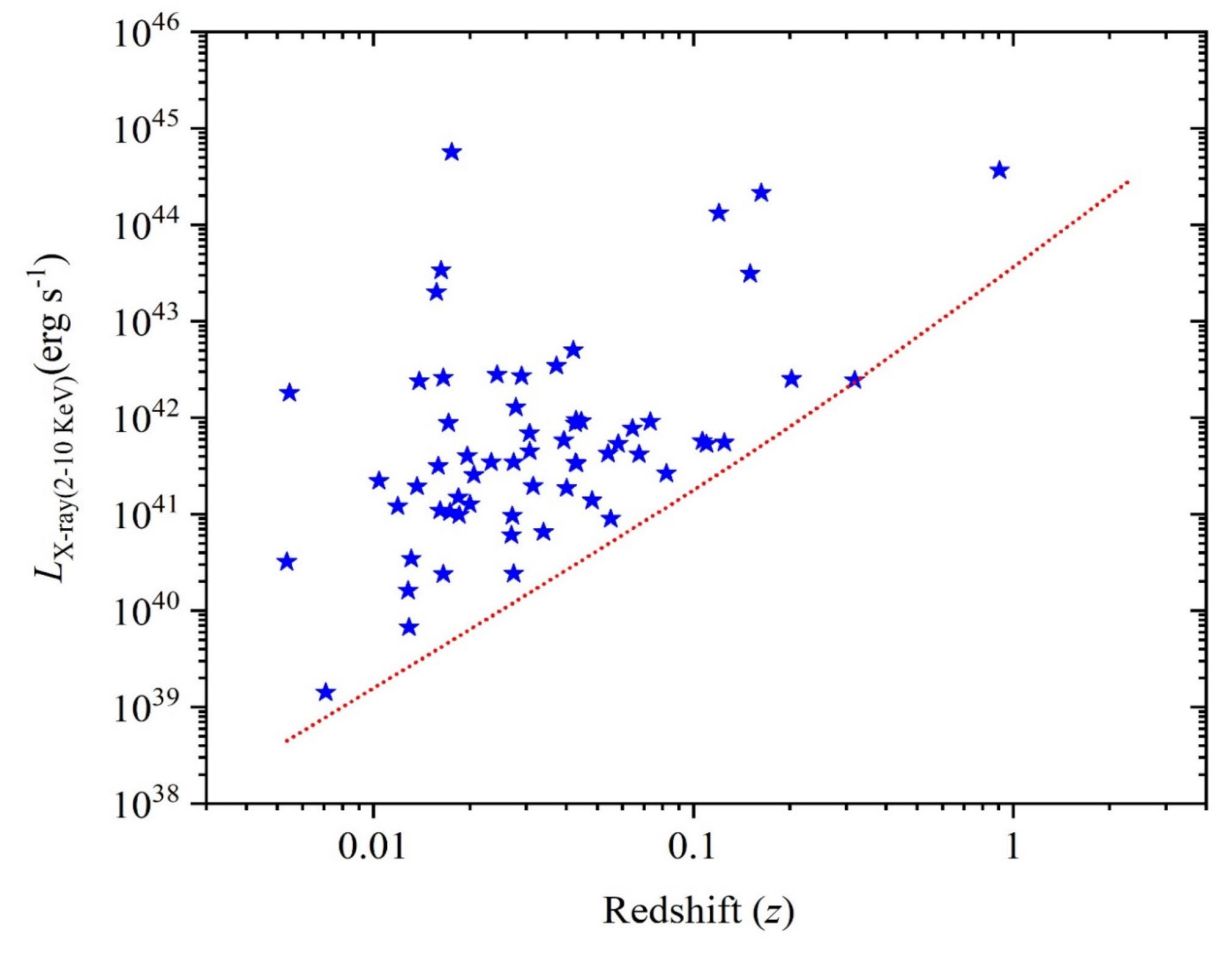
The rest-frame luminosity of 63 sources: the X-ray luminosity in the range of 2–10 keV. The dotted line represents the flux-limited cut-off line considering the Malmquist bias where the minimum fluxes are 7.0 × 10^−15^ erg s^−1^ cm^−2^ for X-ray luminosity.

The sample of galaxies was selected considering the availability of observed X-ray fluxes available from the NED (NASA/IPAC Extragalactic Database) across different energy bands where they carried out using diverse X-ray satellites. Since the sample selection is flux-limited, the sample was tested to the Malmquist bias in relevance to the redshift as shown in Fig. 1 where the rest-frame X-ray luminosity (*L*_*2–10keV*_) was adapted to the 2–10 keV range. The luminosity values were calculated relying on X-ray flux densities (*F*_*X−ray*_) within the 2–10 keV energy band. These values were corrected for the galactic absorption by approximately a factor of 2 using PIMMS (Portable Interactive Multi-Mission Simulator;^78^ which is related to the hydrogen column density. The rest-frame *L*_*2–10keV*_ was estimated by *L*_*2–10keV*_*= 4π*(*d*_*L*_)^*2*^*F*_*2–10keV*_, where *d*_*L*_ is the luminosity distance. The *K*-correction for this luminosity was carried out as the same way in Appendix B in^79^.

Across the multiwavelength of the SED, observational data were extracted from the NED in mJy. In the X-ray band, flux densities (*F*_*X−ray*_) within the 2–10 keV energy band are considered. In UV/optical/IR ranges, flux measurements were sourced from photometric instruments on various telescopes, including GALEX, SDSS, 2MASS, Spitzer, IRAS, and Herschel where each is equipped with different filter. The X-CIGALE code has prepared to configure with the set of filters. Flux densities in the UV bands were obtained from GALEX, while those in the *ugriz* bands were sourced from SDSS. The NIR bands (J, H, and K) fluxes were extracted from 2MASS, and the Infrared Array Camera (IRAC) bands at 3.6 and 4.5 μm were derived from Spitzer. In the MIR range, fluxes at 5.8 and 8 μm, as well as the Multiband Imaging Photometer for Spitzer (MIPS) band at 24 μm, were obtained from Spitzer. The 24 μm band data were also taken from IRAS. For the FIR bands, fluxes were gathered from IRAS for the 24 μm band, and from Herschel, using the Photodetector Array Camera and Spectrometer (PACS), for bands at 70, 100, and 160 μm. Additionally, data for bands at 450 and 850 μm were sourced from SCUBA. For all selected sources, each one has photometric measurements across the optical, NIR, MIR, and far-IR regimes, in addition to the X-ray band.

The X-CIGALE code was conducted to reproduce SED models for all galaxies of the sample. The best fit of these models, controlled by the free parameters given in Table 1, is evaluated by getting best values of the reduced chi-square (*χ*^2^) where its values are listed in the Table A1 (Appendix A).

## Results and analysis

**Fig. 2:**
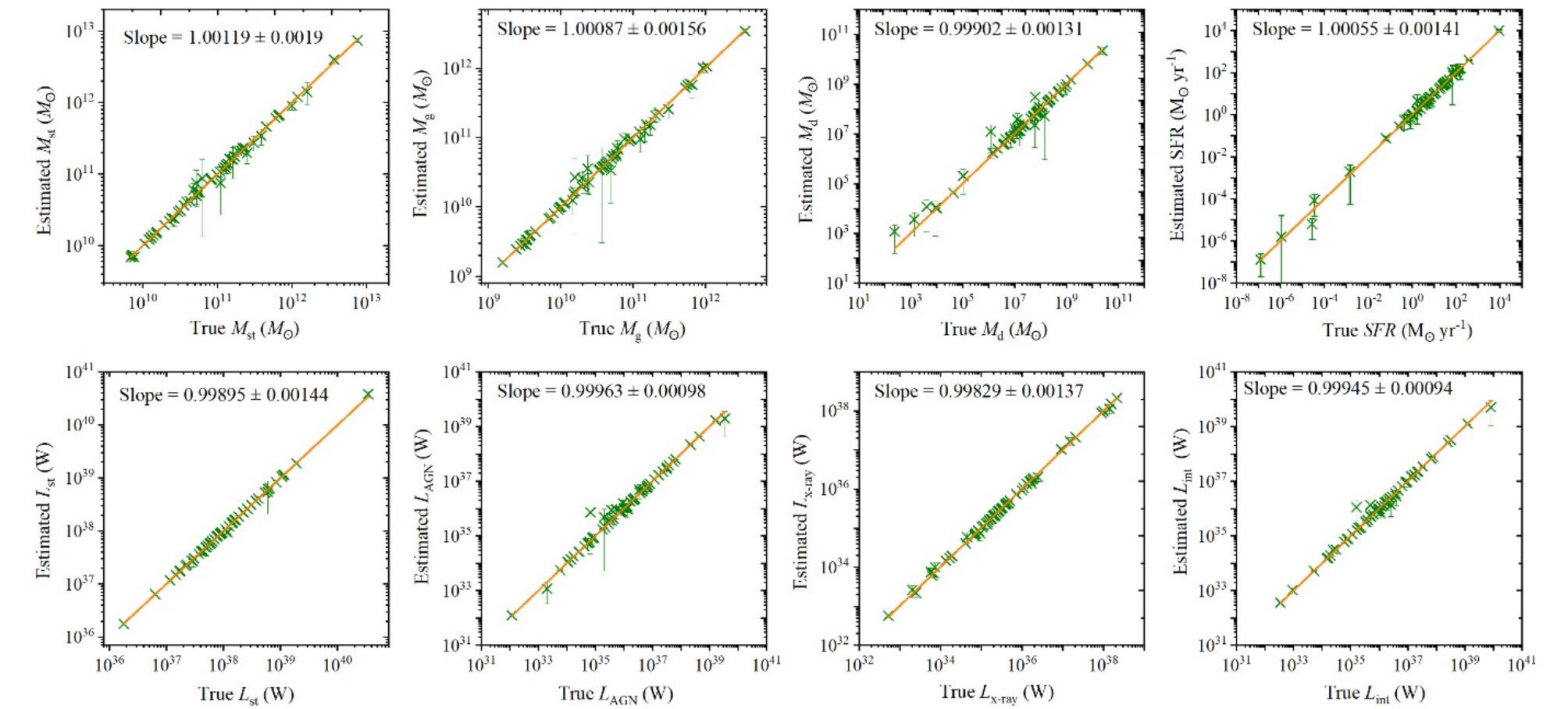
The relationship between estimated and true values (mock results) is depicted for *M*_st_, *M*_g_, and *M*_d_, SFR in the top panels (from left to right) and for the luminosity components *L*_st_, *L*_AGN_, *L*_X-ray_, and *L*_int_ in the bottom panels (from left to right), and vertical lines indicate the 1σ error bars.

For the sample of U/LIRGs, the SED curves were fitted with observational data to estimate various galactic physical parameters. The findings, generated by X-CIGALE code, are possibly affirmed by the analysis of a mock catalogue which serves as a consistent method giving the reliability of the derived physical properties. In this approach, the code determines the best fit for each object, constructing a mock catalogue. For each best-fit flux, noise is introduced by adding random values drawn from a Gaussian distribution with the same standard deviation as the observed flux. This mock data is analyzed in the same manner as the original observed data, allowing the accuracy of the parameter estimation to be evaluated by comparing observed values with the outputs of estimated values. The mock results that compare the estimated to observed physical parameters are shown in Fig. 2. The upper panels display stellar, gas, and dust masses (*M*_st_, *M*_g_, and *M*_d_, respectively) and the star formation rate (SFR). The lower panels illustrate the luminosity components which include stellar luminosity (*L*_st_), AGN luminosity (*L*_AGN_), X-ray AGN luminosity (*L*_X−ray_), in addition to the intrinsic (unextinct) AGN luminosity (*L*_int_) which represents the AGN accretion power. All these parameters demonstrate strong consistency between the estimated and observed values, as evidenced by their correlated relationship. The reliability of these estimates is indicated by the slope’s value in the insets of these panels where the slope approaches the unity.

**Fig. 3:**
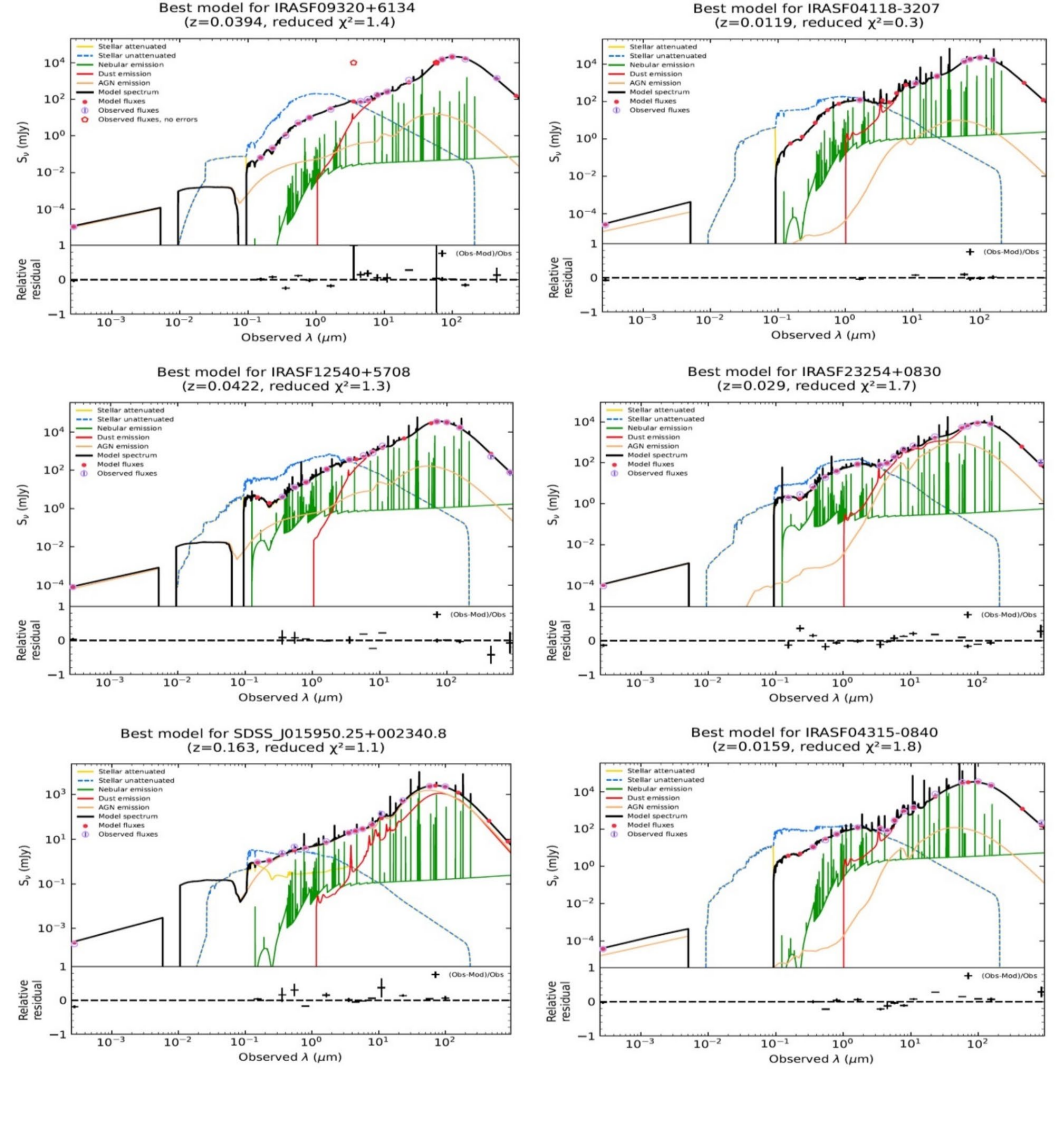
SED models of IRASF04118-3207, IRASF23254 + 0830 and IRASF04315-0840 as examples of LIRG (*right panels*), and IRASF09320 + 6134, IRASF12540 + 5708 and SDSS_J015950.25 + 002340.8 as examples of ULIRG galaxies (*left panels*). The SED model spectrum is displayed with various components: attenuated stellar emission (*yellow line*) and unattenuated stellar emission (*dashed blue line*), nebular emission (*green line*), dust emission (*red line*), and AGN emission (*orange line*). The estimated model flux densities are represented by *red dots*, while the observed flux densities, along with their error bars, are denoted by *open circles* in *light magenta*.

For some selected sources from the galaxy sample, Fig. 3 shows the SED fittings, in the rest frame, performed using the X-CIGALE code. The SED spectra of U/LIRGs, characterized by their IR emissions, clearly highlight differently the contribution of emissions overall multiwavelength spectra from the X-ray to FIR range.

### Mass and SFR-luminosity dependences

**Fig. 4:**
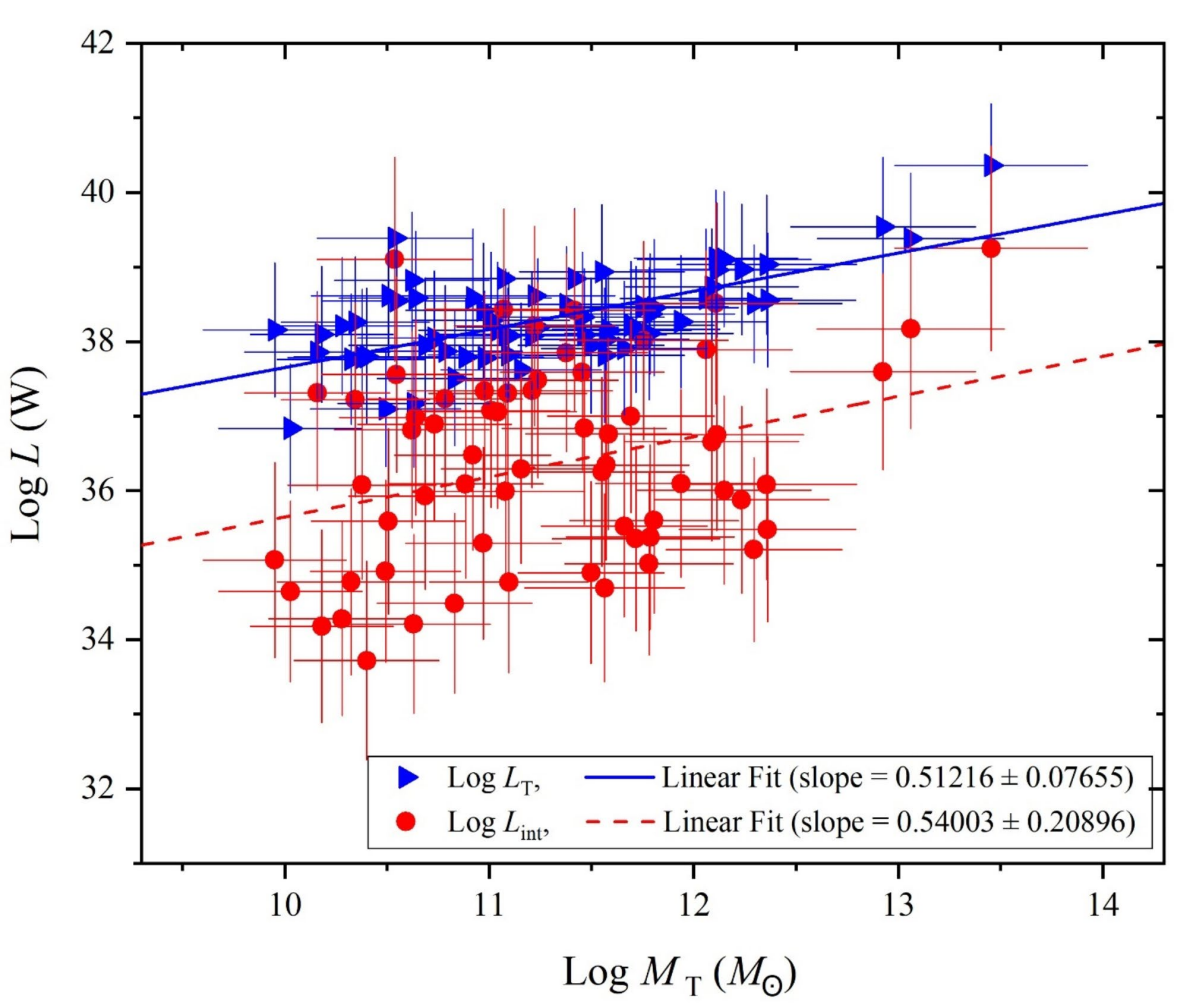
The total and intrinsic luminosities versus the galaxy mass of U/LIRGs.

In this subsection, we first present the resulted SED physical properties including the mass components and SFR, in relations with the decomposed (stellar, AGN, X-ray) luminosities of U/LIRGs. For which, the variation of these luminosities is generally described versus the total and stellar masses, and the SFR. For the host galaxy disk linked with its accretion disk’s power, Fig. 4 presents, in a logarithmic scale, the variations of the total luminosity including stellar, AGN and X-ray luminosities of the galaxy, and those of the intrinsic AGN luminosity with increasing the total mass (*M*_T_) including star, gas and dust masses of that galaxy. These variations display a notable increase in their general trend profiles with averaged values of their slopes of 0.51216 ± 0.07655 and 0.54003 ± 0.20896 for *L*_T_ and *L*_int_, respectively. This indicates that unabsorbed total luminosity and the intrinsic AGN luminosity are strongly associated with increasing the total mass.

**Fig. 5:**
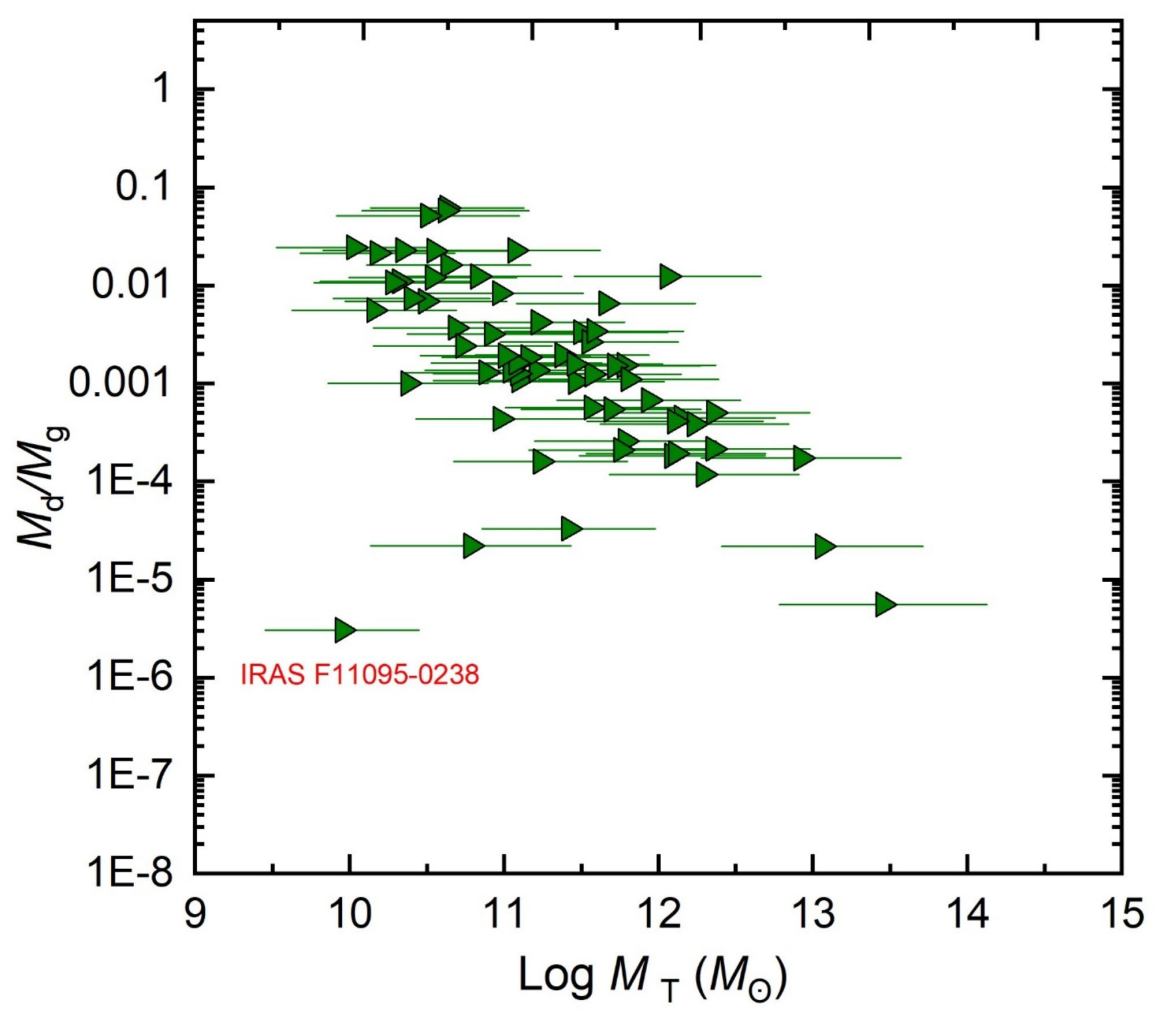
Logarithmic variation of the dust-to-gas mass ratio versus the total galaxy mass.

Within the host galaxy disk, the luminosity, partially absorbed by dust, dominates the SED spectrum in the IR ranges. This is mainly attributed to the re-emitted AGN dust luminosity where the U/LIRGs are characterized by their luminous IR emissions. For the dust-to-gas mass ratio (*M*_d_/*M*_g_) of the galaxy sample of U/LIRGs, Fig. 5 illustrates that this ratio decreases with increasing *M*_T_, varying from ∼ 0.06 at the highest mass to ∼ 5.6 × 10^−6^ at the lowest one. Only one object (IRASF11095-0238) deviates from this pattern, displaying a lower value at low mass.

**Fig. 6:**
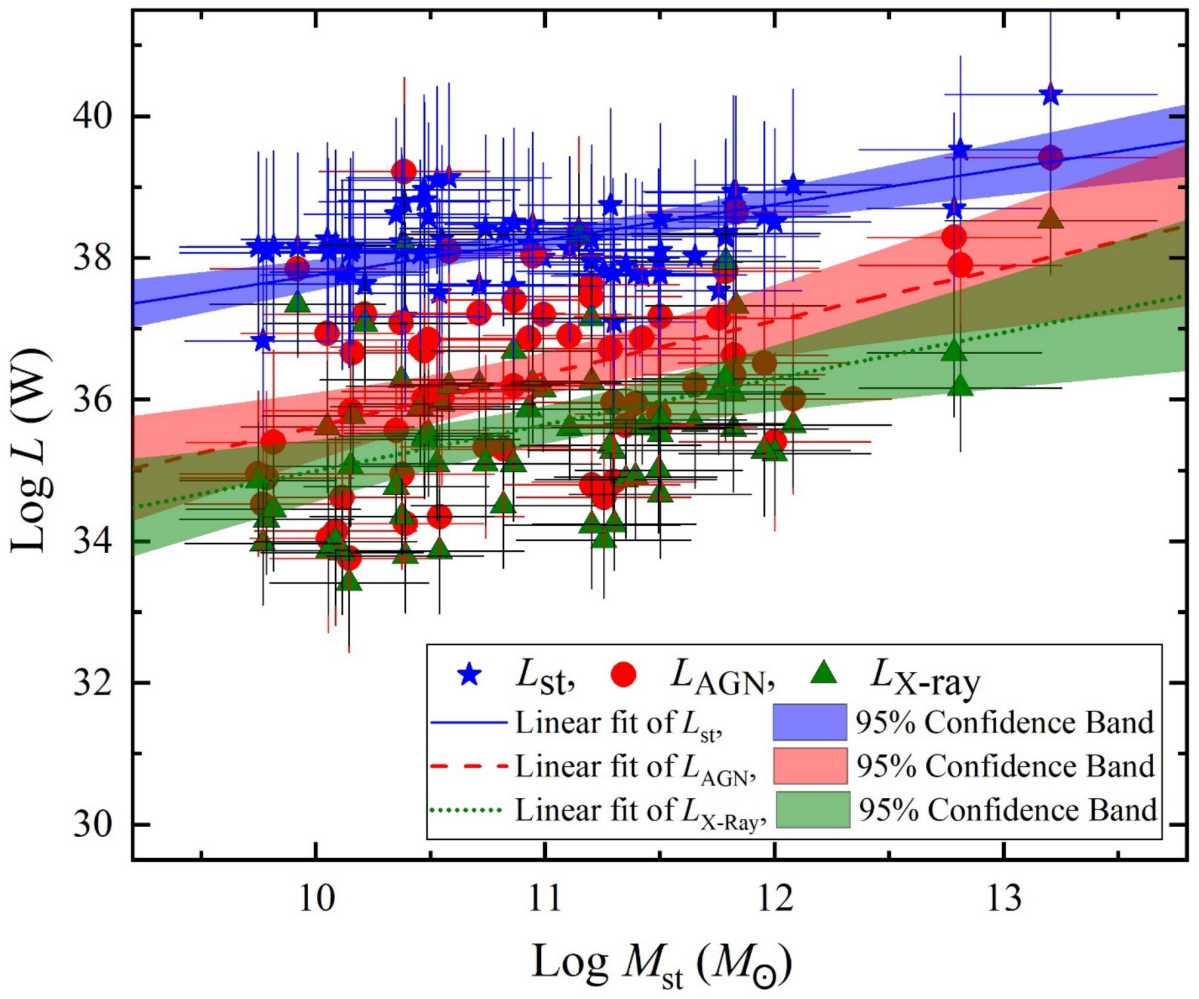
The luminosity components of stellar luminosity (blue stars), AGN luminosity (red circles), and x-ray luminosity (green triangles) versus stellar mass.

**Fig. 7:**
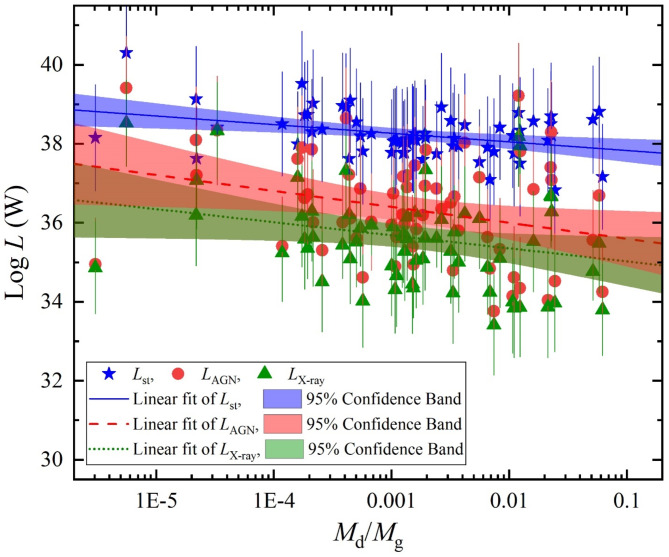
The luminosity components of stellar luminosity (blue stars), AGN luminosity (red circles), and x-ray luminosity (green triangles) versus dust to gas mass ratio.

With decomposing the total luminosity over its SED curve from the X-ray to the FIR, essential components of *L*_st_, *L*_AGN_ and *L*_x−ray_ are produced. Figures 6 and 7 display the variation of these luminosity components versus *M*_st_ and *M*_d_/*M*_g_, respectively. For which, the *L*_st_ largely dominates the luminosity emissions while both of *L*_AGN_ and *L*_X−ray_ have a smaller contribution. In Fig. 6, the linear fit *L*_st_, *L*_AGN_ and *L*_x−ray_ with 95% confidence band of their logarithmic values shows positive various variations with increasing the stellar mass, giving a Pearson-r coefficient of 0.43, 0.44, and 0.42, respectively. On the other hand, as shown in Fig. 7, the variations these luminosities versus the logarithm of dust-to-gas mass ratio have a passive variation with Pearson-r coefficient of -0.37, -0.29, and − 0.26, respectively.

.

**Fig. 8:**
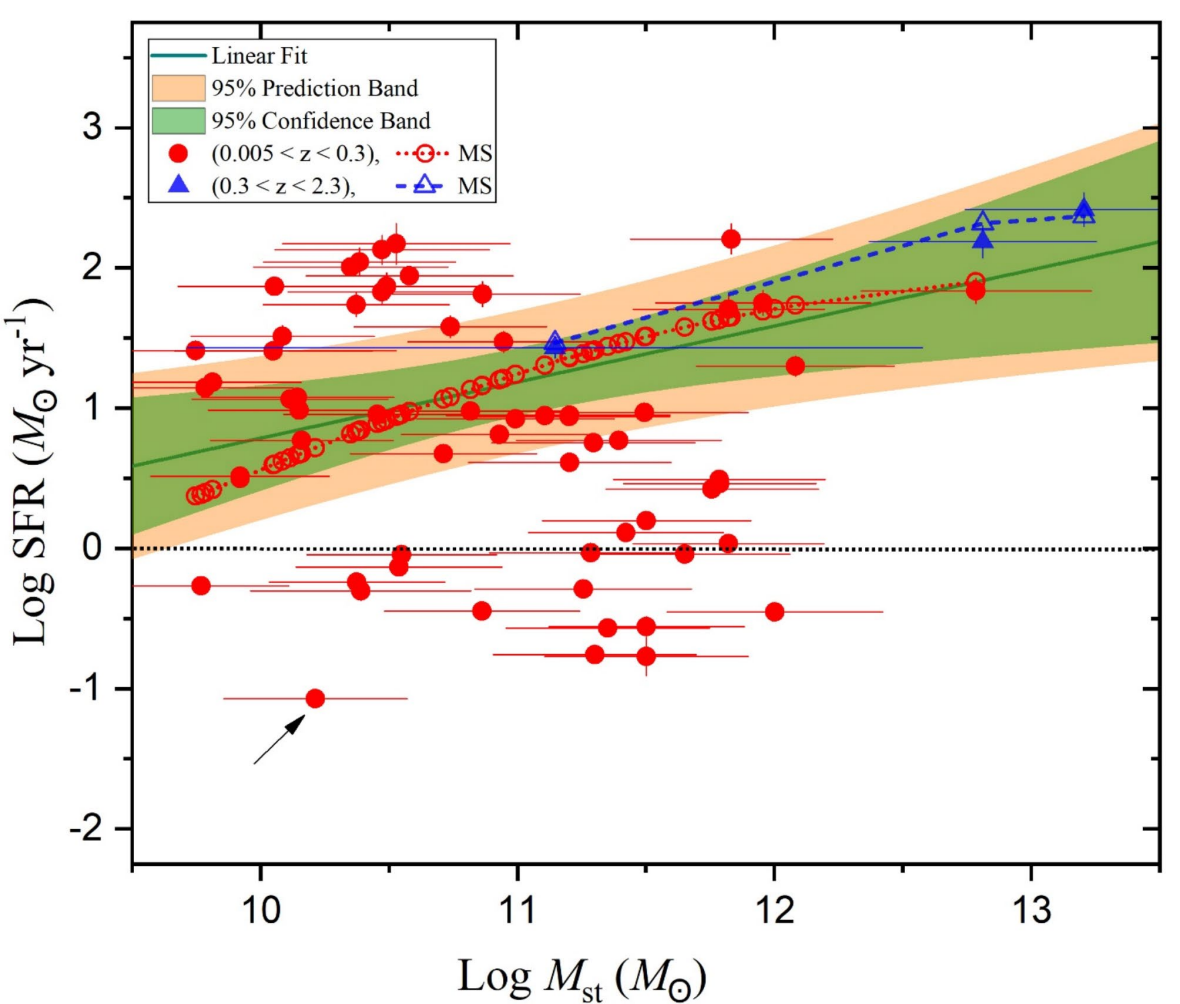
The variation SFR versus the stellar mass of U/LIRG galaxies. Below the dotted horizontal line there are 15 galaxies having values of SFR less than 1.

According to the mass-luminosity characterization mentioned above, we can also induce the SFR-luminosity dependence of U/LIRGs which is linked to mass variation. Figure 8 illustrates that the trend variation of the SFR generally increases by increasing the stellar mass from ∼5.58 × 10^9^*M*_☉_ to 1.6 × 10^13^*M*_☉_, with a mean value of 〈*M*_st_〉 = 6.43 × 10^11^*M*_☉_. Some samples at intermediate stellar masses with low SFR have a remarkable deviation. The SFR varies from ∼ 0.09 *M*_☉_yr^−1^ for IRASF14544-4255 (with small redshift of 0.01573) to 260.95 *M*_☉_yr^−1^ for IRASF10214 + 4724 (with high redshift of 2.2856), with mean value of 〈SFR〉 = 32 *M*_☉_yr^−1^. For this variation, the linear fit reveals that the averaged SFR increases with *M*_st_, especially for high values of the SFR.

The SFR–*M*_*st*_ relation generally represents the galaxy main sequence (MS) of star formation in galaxies^80–82^. It is shown in Fig. 8 as line-open symbols, calculated using Eq. 5 in^80^with free parameters of the maximum log SFR (S_0_), the turnover mass (M_0_) in log *M*_st_, and the power-law slopes of *α* and *β* at low and high stellar masses, respectively. The adaptation values of these parameters are given in Table A2 in Appendix A. For sources localized within the prediction band of the fit in Fig. 8, there are an agreement with two profiles of the MS provided with ranges of redshifts of 0.005 < *z* < 0.3 (dotted red line-symbol) and another of 0.3 < *z* < 2.3 (dashed blue line-symbol), respectively. On the other hand, there are two groups of sources showing deviation from the fit area. One is above the fit at low masses (*M*_st_ < 10^11^*M*_☉_) while another is low the fit with intermediate masses of *M*_st_ ∼ 10^10^ − 10^12^*M*_☉_. As our sample is for the U/LIRGs, it may contain sources with high SFR and others with low SFR. Sources, which appear above the prediction band of the fit with very high SFR, are probably starburst galaxies due to the high activity of their SFR. Other sources, which appear below the prediction band of the fit with very low SFR, are probably quiescent. The latter are expected to be exposed to galaxy quenching mechanisms such as: *i*) A strong jet, from an active supermassive black holes (SMBH), heats up the cold gas within the galaxy causing a reduction in its star formation activity^83–85^; *ii*) Although the galaxy mergers cause rapid bursts of star formation, hence they produce a high rate of supernovae which hold up the cold gas, leading to quenching^86,87^; *iii*) Environmental quenching occurs because a galaxy is affected by ram pressure force in dense intergalactic medium, leading to gas depletion^88^.

**Fig. 9:**
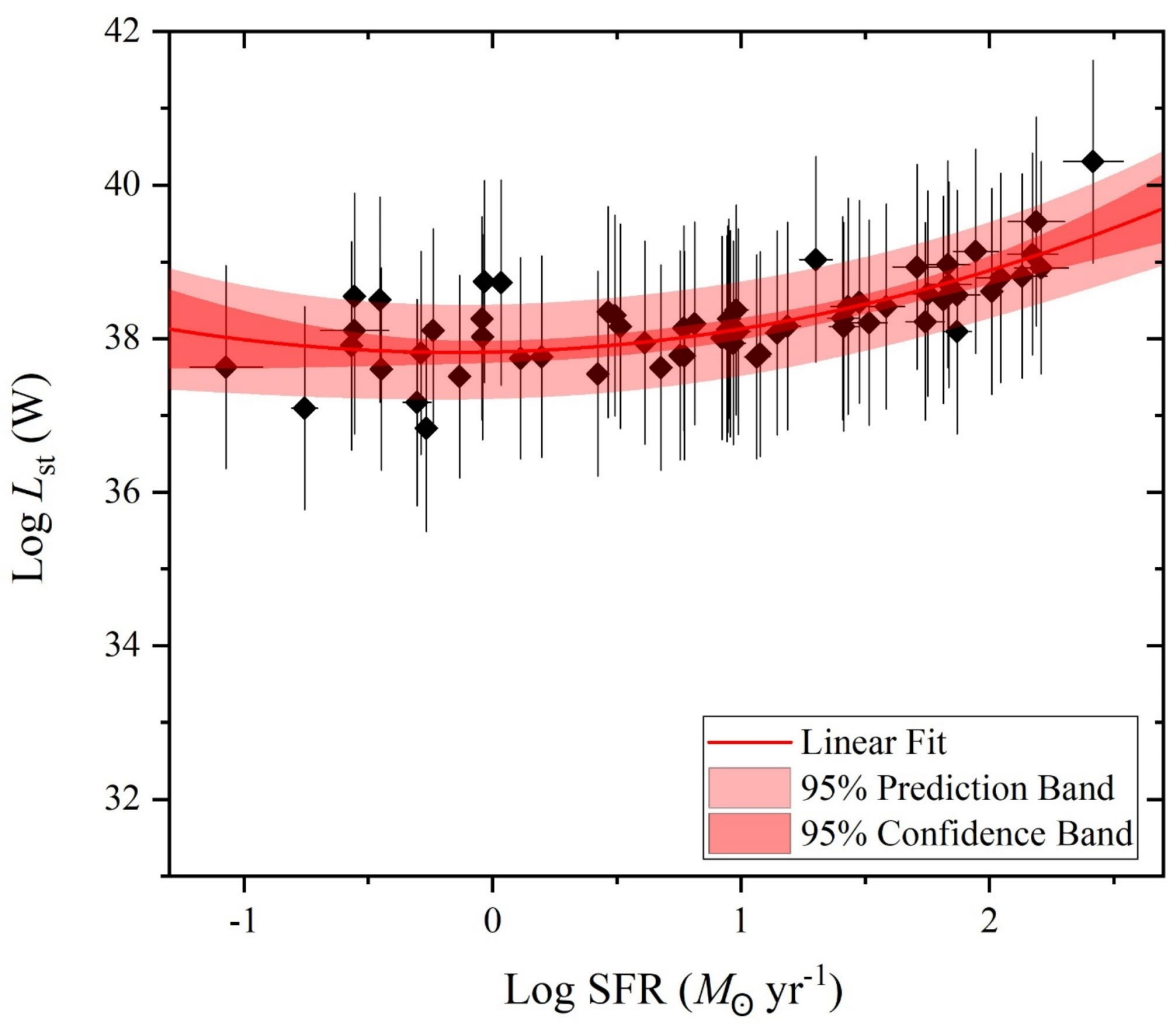
The stellar luminosity versus the SFR.

**Fig. 10:**
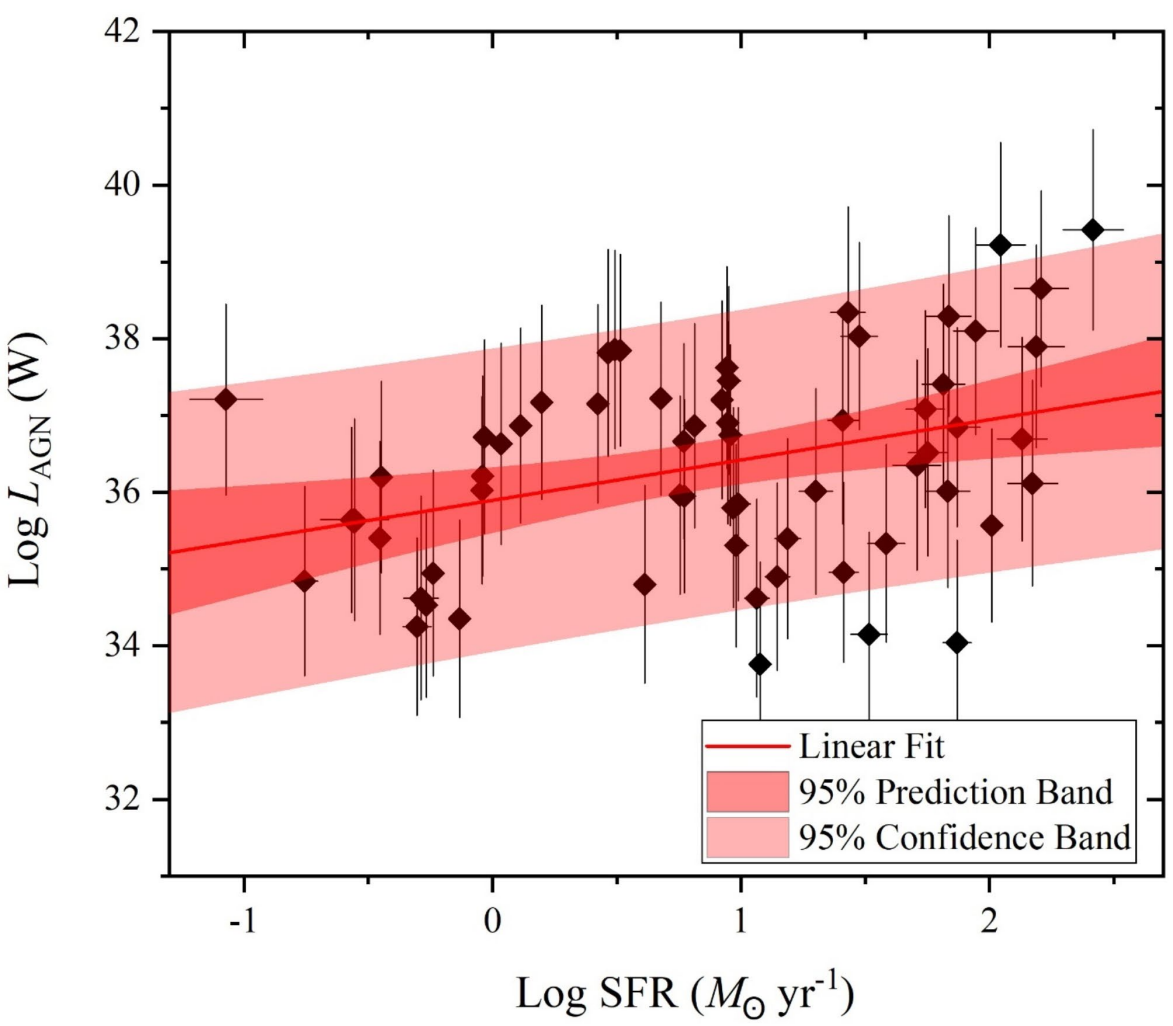
The AGN luminosity versus the SFR.

**Fig. 11:**
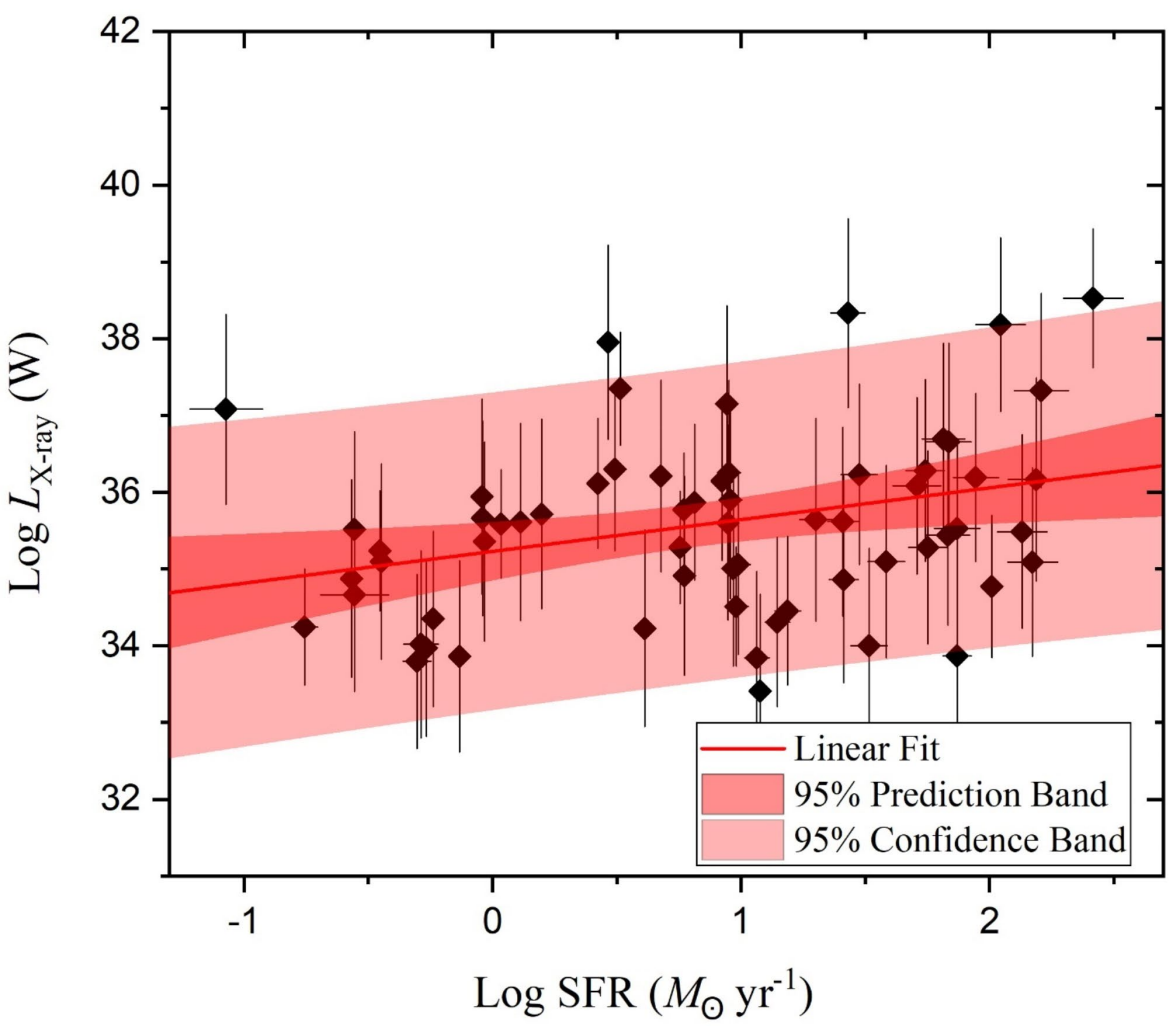
The X-ray luminosity versus the SFR.

Over the considerations of the stellar mass and the SFR variations of our selected galaxy sample of U/LIRG, each *L*_st_, *L*_AGN_ and *L*_x−ray_ can be separately characterized with SFR. In the logarithmic SFR-luminosity frame, dependencies of *L*_st_, *L*_AGN_ and *L*_x−ray_ are shown in Figs. 9, 10 and 11, respectively. For *L*_st_ - SFR relation shown in Fig. 9, the 2nd ordered polynomial fit shows a nearly flat relation at log SFR < 0 for quiescent sources below MS, presented in Fig. 8, and an exponential increase at log SFR > 0 for sources within or above this MS. Both *L*_AGN_ – SFR and *L*_x−ray_ - SFR relations as shown in Figs. 10 and 11, respectively, show increasingly linear variations with a Pearson-r coefficient of 0.36 and 0.32, respectively.

### Analyzed correlations of SED luminosity components

For our results, the correlation coefficient (*r*) of the relationships among the physical properties is calculated. An estimate of a linear correlation has been done in log–log space with x-, y-uncertainties of luminosity-mass and luminosity-luminosity correlations. For these correlations, the Bayesian maximum likelihood method^89^ was used where uniform prior distributions are assumed for the regression parameters. Considering the same way in^35,36,90^, the Markov chain Monte Carlo (MCMC) simulation was conducted using the mean and standard deviation from the posterior probability distributions with 10,000 iterations.

For the results shown in Figs. 4, 6 and 7 in *log-log* frame, the values of the correlation coefficient of the luminosity-mass dependences are listed in Table 2. Under the considerations assumed for the strength of each relationship in this table, we accordingly clarify its correlation coefficient. Versus the total mass shown in Fig. 4, the total luminosity shows a strong correlation while the intrinsic luminosity shows an intermediate one. For the luminosity components shown in Fig. 6 versus *M*_st_, they have a strong correlation with increasing the stellar mass. On the other hand, as shown in Fig. 7, both of *L*_st_, *L*_AGN_ and *L*_X−ray_ have weak correlations with increasing the dust contribution where they slightly decrease.

**Fig. 12:**
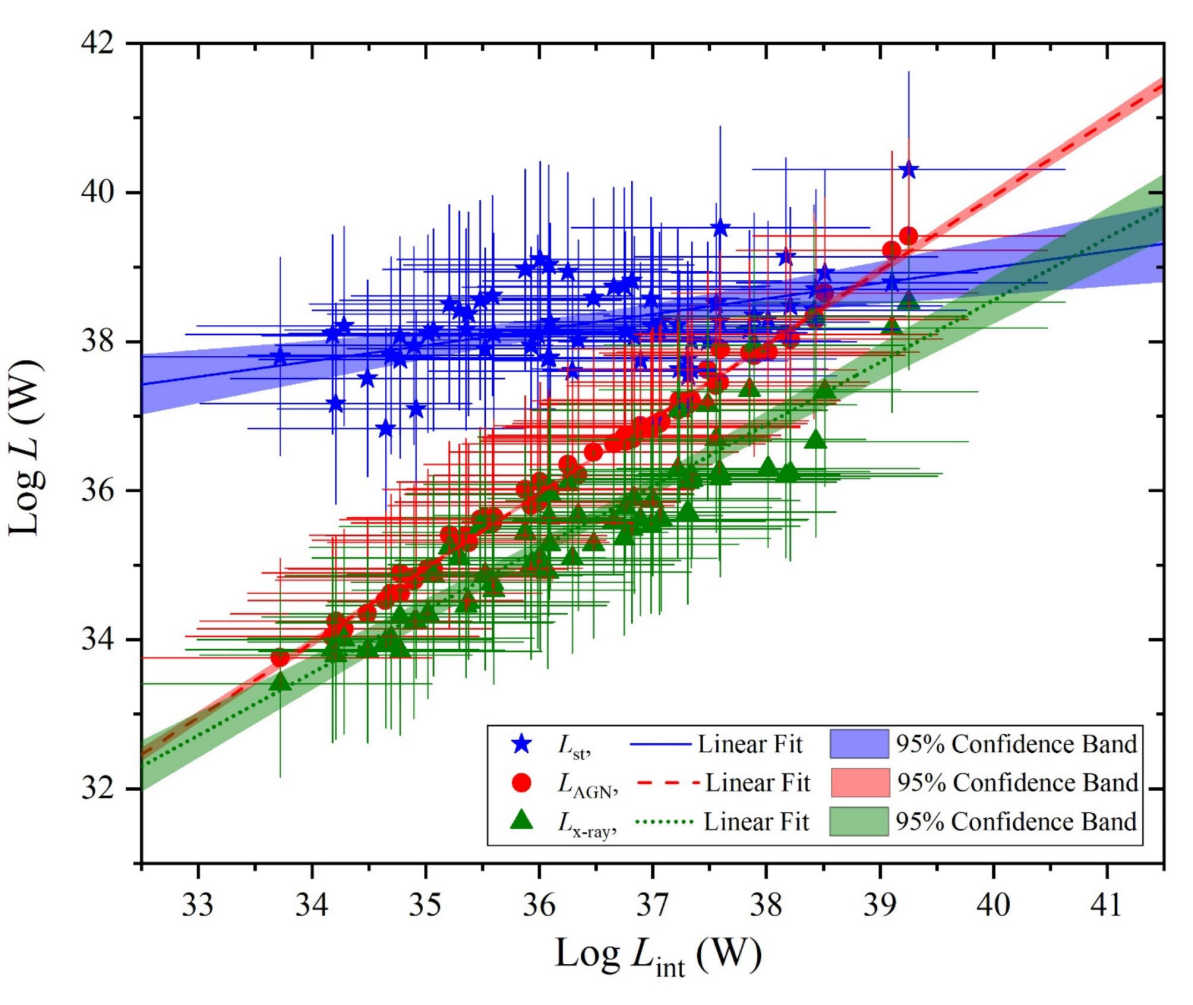
The luminosity components, represented by the AGN luminosity (blue stars), stellar luminosity (red circles), and X-ray luminosity (black triangles) versus the intrinsic AGN luminosity.

In a logarithmic frame of luminosity-luminosity relations as shown in Fig. 12, variations of both of *L*_st_, *L*_AGN_ and *L*_x−ray_ are illustrated as a function of the intrinsic luminosity of the AGN’s disk accretion power. These variations demonstrate upward trends but with different variation rates as the intrinsic AGN luminosity increases. The correlation coefficients of their variations with intrinsic luminosity are also listed in Table 2. It is obviously that these correlation coefficients are strong for U/LIRGs in agreement with those of obscured AGN galaxies^24^ which is characterized by a highly silicate absorption band at 9.7 μm. What is only different is that the *L*_st_ - *L*_int_ correlation is slightly lower for U/LIRG than that of for obscured AGN galaxies.

**Fig. 13:**
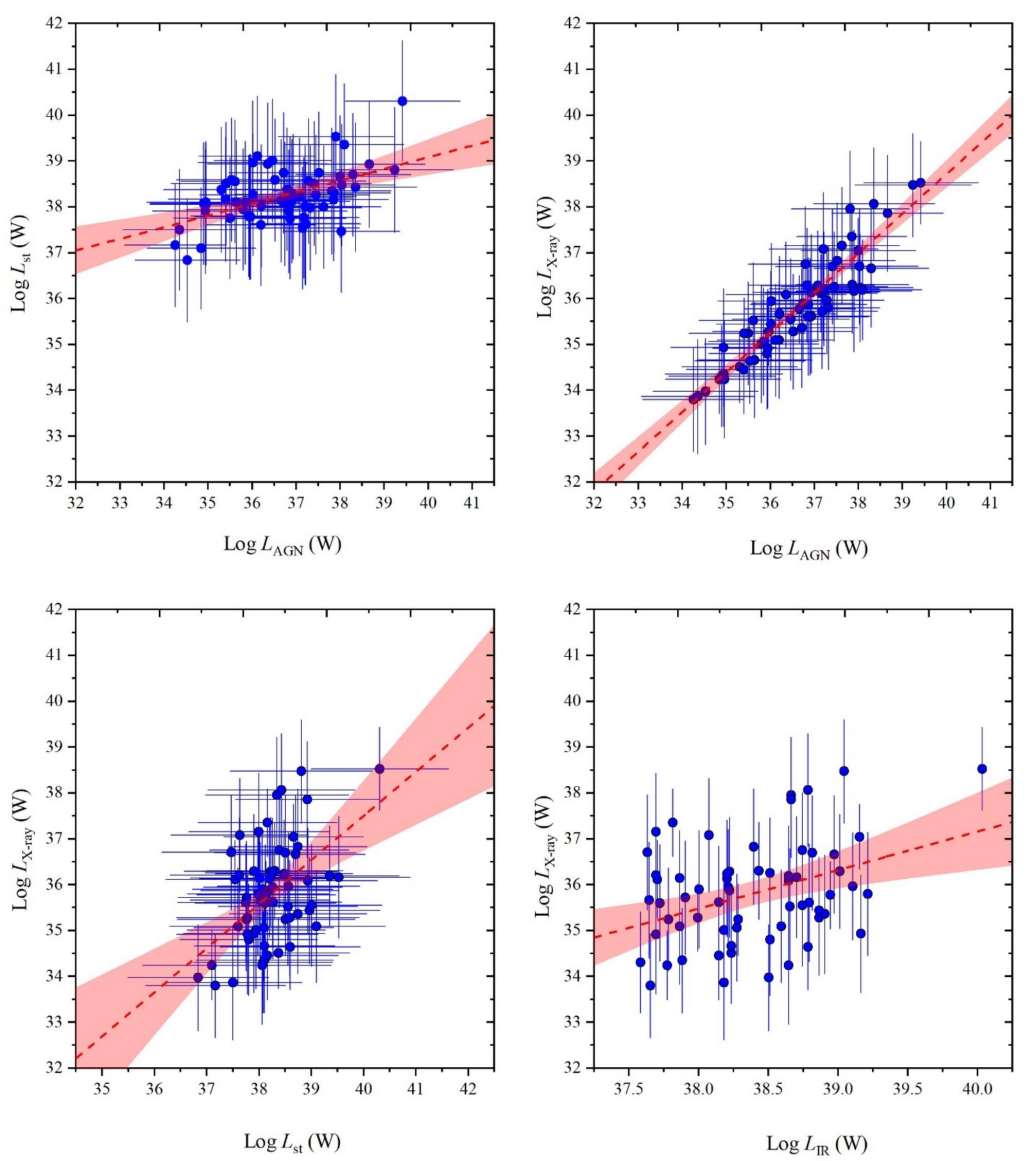
Logarithmic relationships between the luminosity components including *L*_st_ and *L*_x−ray_*versus* the *L*_AGN_ (*top panels*), and *L*_x−ray_ versus *L*_st_ and *L*_IR_ (*bottom panel*), where values of *L*_IR_ were collected from the literature as listed in Table A1 in Appendix A. dashed-lines represents the linear fit with 95% confidence band (red-shaded areas).

Figure 13 displays the relationships between the luminosity components themselves in *log*-*log* frame. From which, the variation of *L*_st_ and *L*_X−ray_ versus *L*_AGN_ (*top panels*) have strong correlation coefficients. As listed in Table 2, they have correlation coefficients with 0.867 ± 0.001 and 0.982 ± 0.001, respectively. On the other hand, those of *L*_X−ray_ versus *L*_st_ has a strong correlation while it versus *L*_IR_ has an intermediate correlation. For *L*_X−ray_ component, its values (in erg s^−1^) and the corresponding AGN fractions (*f*_AGN_) are given in Table A1 in Appendix A. For these parameters, we found that 19 sources from our sample have *L*_X−ray_ < 10^42^ erg s^−1^ where their SEDs fit with very low AGN fraction (< 0.02) with an exception for only one source (IRASF13197-1627) which its SED fits with high AGN fraction (0.35). This source may exhibit a strong AGN power originating from the SMBH.

**Table 2:** The correlation coefficients of the relationships between the SED properties.

Relationship	U/LIRGs current work		Obscured galaxies^a^	
	*r* ^b^	s^c^	*r*	s
*Log L*_T_ − *Log M*_T_	0.906 ± 0.001	✓	……	……
*Log L*_int_ − *Log M*_T_	0.355 ± 0.005	•	……	……
*Log L*_st_ − *Log M*_st_	0.679 ± 0.003	✓	……	……
*Log L*_AGN_ − *Log M*_st_	0.510 ± 0.004	✓	……	……
*Log L*_X−ray_ − *Log M*_st_	0.764 ± 0.002	✓	……	……
*Log L*_st_ − *M*_d_/*M*_g_	-0.017 ± 0.007	−	……	……
*Log L*_AGN_ − *M*_d_/*M*_g_	-0.017 ± 0.005	−	……	……
*Log L*_X−ray_ − *M*_d_/*M*_g_	-0.020 ± 0.006	−	……	……
*Log L*_st_ − *Log L*_int_	0.836 ± 0.001	✓	0.969 ± 0.001	✓
*Log L*_AGN_ − *Log L*_int_	0.982 ± 0.001	✓	0.970 ± 0.001	✓
*Log L*_X−ray_ − *Log L*_int_	0.983 ± 0.001	✓	0.940 ± 0.001	✓
*Log L*_st_ − *Log L*_AGN_	0.867 ± 0.001	✓	……	……
*Log L*_X−ray_ − *Log L*_AGN_	0.982 ± 0.001	✓	……	……
*Log L*_X−ray_ − *Log L*_st_	0.648 ± 0.003	✓	……	……
*Log L*_X−ray_ − *Log L*_IR_	0.357 ± 0.005	•		

Note. ^***a***^Gadallah, 2023. ^***b***^The correlation coefficient (*r*). ^***c***^The strength of the correlation coefficient where | *r* | ≥ 0.5, 0.5 > | *r* | ≥ 0.1, and | *r* | < 0.1 represent a strong (✓), intermediate (•), and weak (−) strength, respectively.

## Discussion

In comparison with other selected U/LIRG sample (67 galaxies) collected from GOALS, recent study by^91^ reproduced the SED fittings in a multi-wavelength range from the UV to the sub-millimetre. In which, the values of SFR have higher range from 2.4 to 410 *M*_☉_yr^−1^ with 〈SFR〉 = 81 *M*_☉_yr^−1^ than those of our sample (63 galaxies) which varies from ∼ 0.09 *M*_☉_yr^−1^ to 260.95 *M*_☉_yr^−1^ with 〈SFR〉 = 32 *M*_☉_yr^−1^. For our sample presented in Fig. 8, it is obvious that 15 galaxies laying below the dotted horizontal line have values of SFR less than 1 as listed bottom in Appendix A. For these sources, their corresponding AGN fractions are very low (< 0.06) with an exception for one source (IRASF14544-4255; referred by an arrow in Fig. 8) where its AGN fraction is high (0.3) giving high *L*_X−ray_ of 1.2 × 10^44^ erg s^−1^. This source has the lowest value of the SFR limits with low mass of ≈ 1.63 × 10^10^*M*_☉_.

**Fig. 14:**
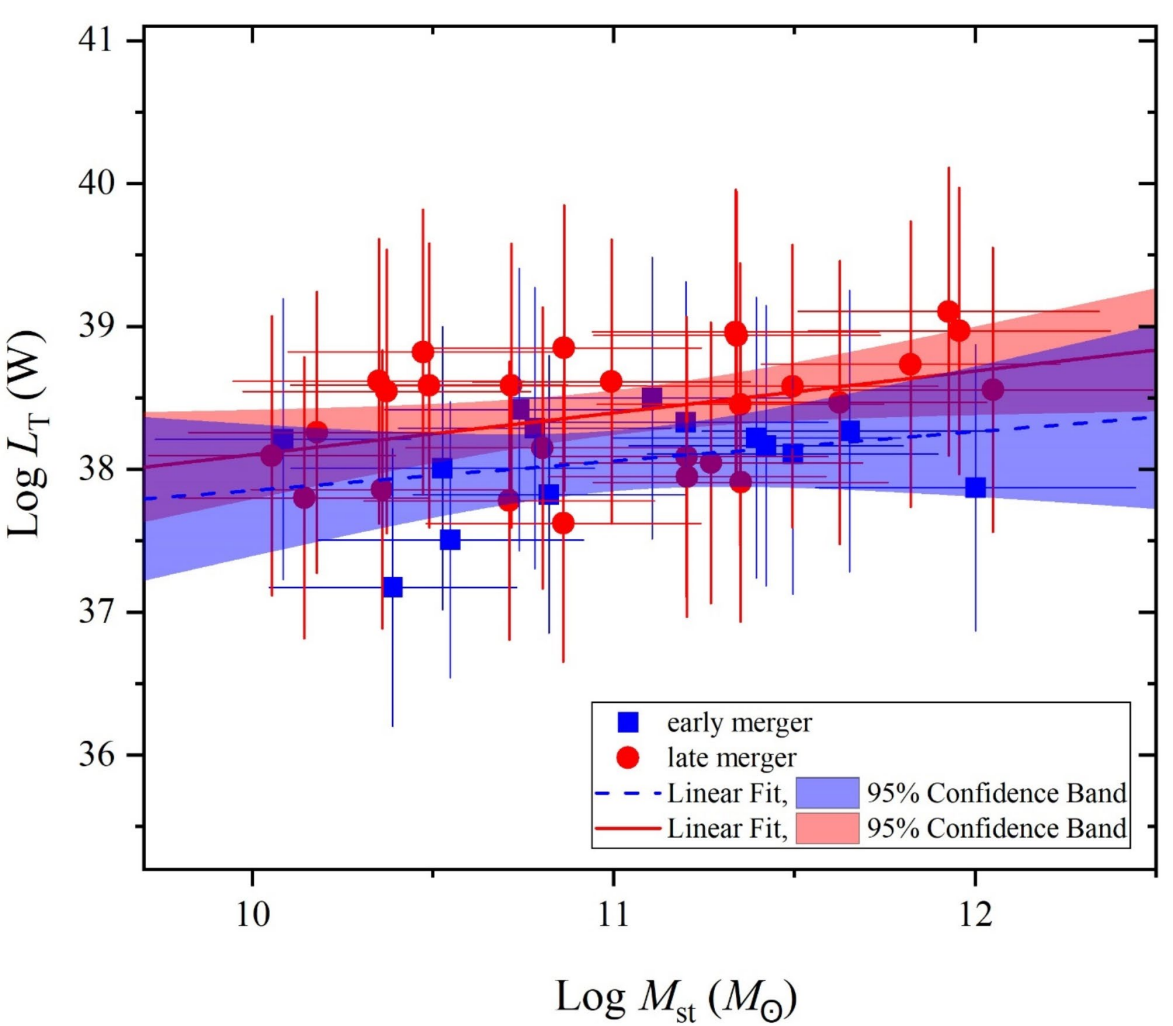
The total luminosity versus the stellar mass of samples classified as early (blue squares) and late mergers (red circles) as classified by^41^.

For selected samples classified as early and late mergers (Appendix A) based on the classification by^41^, Fig. 14 also displays a remarkable increase of their total luminosity with increasing the stellar mass. Obviously, both early and late mergers are scattered and distributed through the mass variation scale from ∼10^10^*M*_☉_ to ∼10^12^*M*_☉_, confirming that their classification is independent of the stellar mass. This is consistent with^41^ where the averaged values of logarithmic *M*_st_ doesn’t change with the merger stages. The linear fits show that the averaged total luminosity of late mergers is higher than that of early ones by a factor of 1.7 at the lowest stellar mass (∼10^10^*M*_☉_) and 2.5 at the highest one (∼10^12^*M*_☉_). On the other hand, these authors found that the SFR is effective where it increases with the merger stages.

In relevance to the variation of the luminosity components as a function of the intrinsic luminosity, the evolution of their trend profiles shown in Fig. 12 can be expressed by the following relationships:

4 $$\:\text{log}({L}_{\text{s}\text{t}})=\left(0.21\pm\:0.5\right)\:\times\:\:\text{l}\text{o}\text{g}({L}_{\text{i}\text{n}\text{t}})\:+\:\:30.61\pm\:1.79\:$$

5 $$\:\text{log}({L}_{\text{A}\text{G}\text{N}})=\left(0.99\pm\:0.01\right)\:\times\:\:\text{l}\text{o}\text{g}({L}_{\text{i}\text{n}\text{t}})\:+\:\:0.02\pm\:0.39\:$$

6 $$\:\text{log}({L}_{\text{X}-\text{r}\text{a}\text{y}})=\left(0.83\pm\:0.04\right)\:\times\:\:\text{l}\text{o}\text{g}({L}_{\text{i}\text{n}\text{t}})\:+\:\:5.19\pm\:1.52\:$$

These relationships indicate that both AGN and X-ray (in the interval of 2–10 KeV) emissions are strongly corelated to the intrinsic accretion power that fuels the host galaxy disk than those of the stellar emissions from stars.

**Fig. 15:**
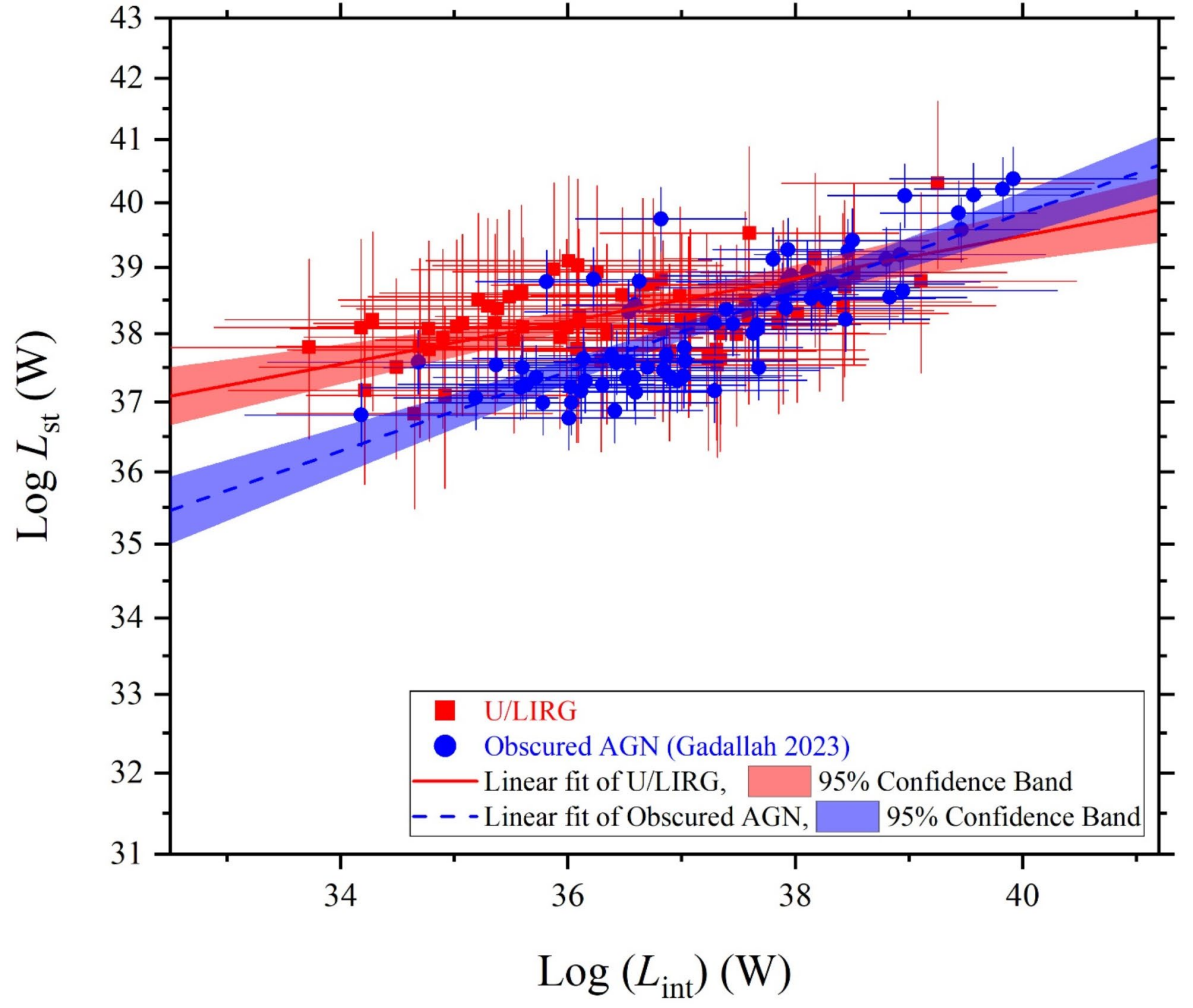
The stellar luminosity versus the intrinsic luminosity of U/LIRGs compared with those of obscured AGN galaxies^24^.

**Fig. 16:**
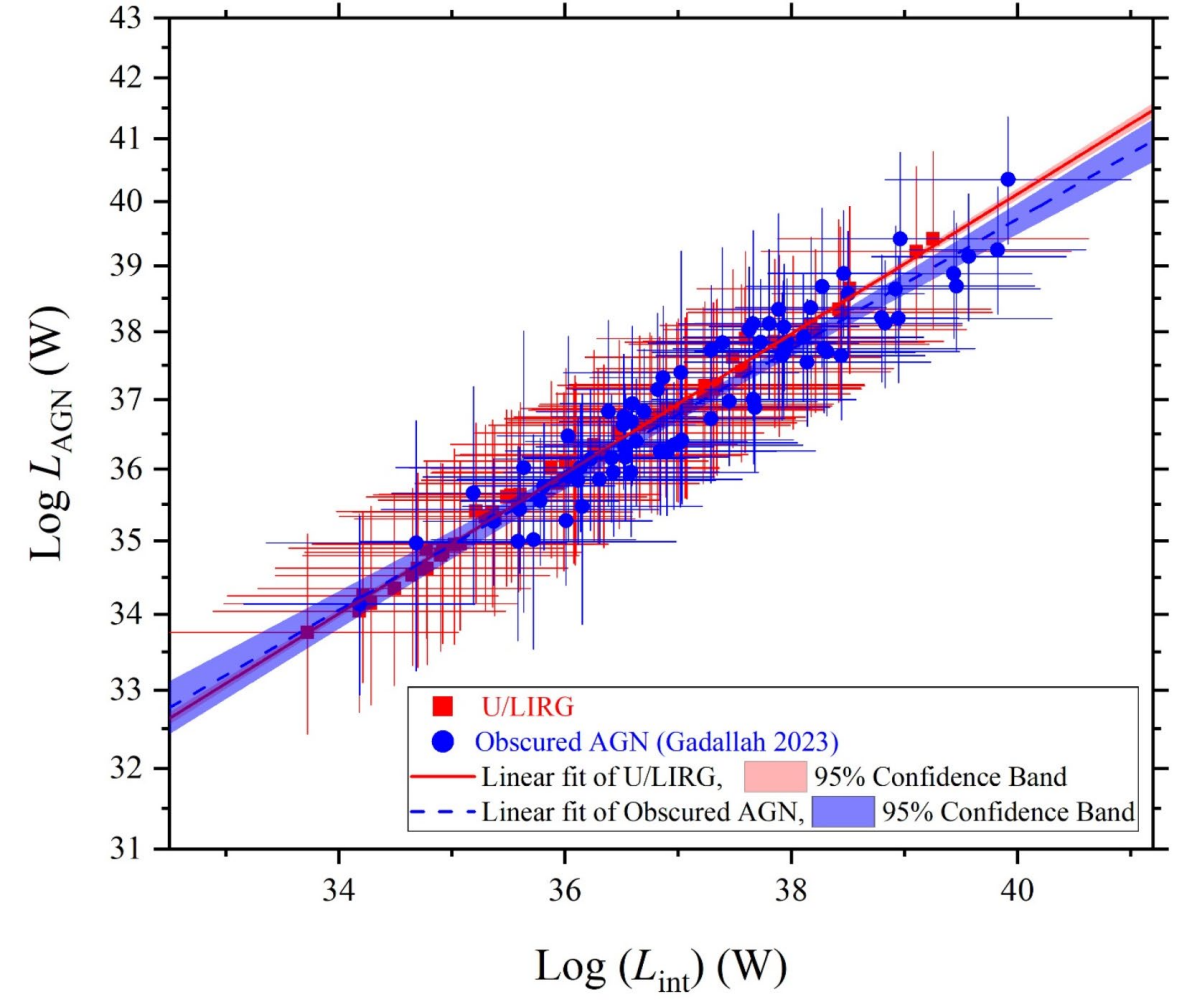
The AGN luminosity versus the intrinsic luminosity of U/LIRGs compared with those of obscured AGN galaxies^24^.

**Fig. 17:**
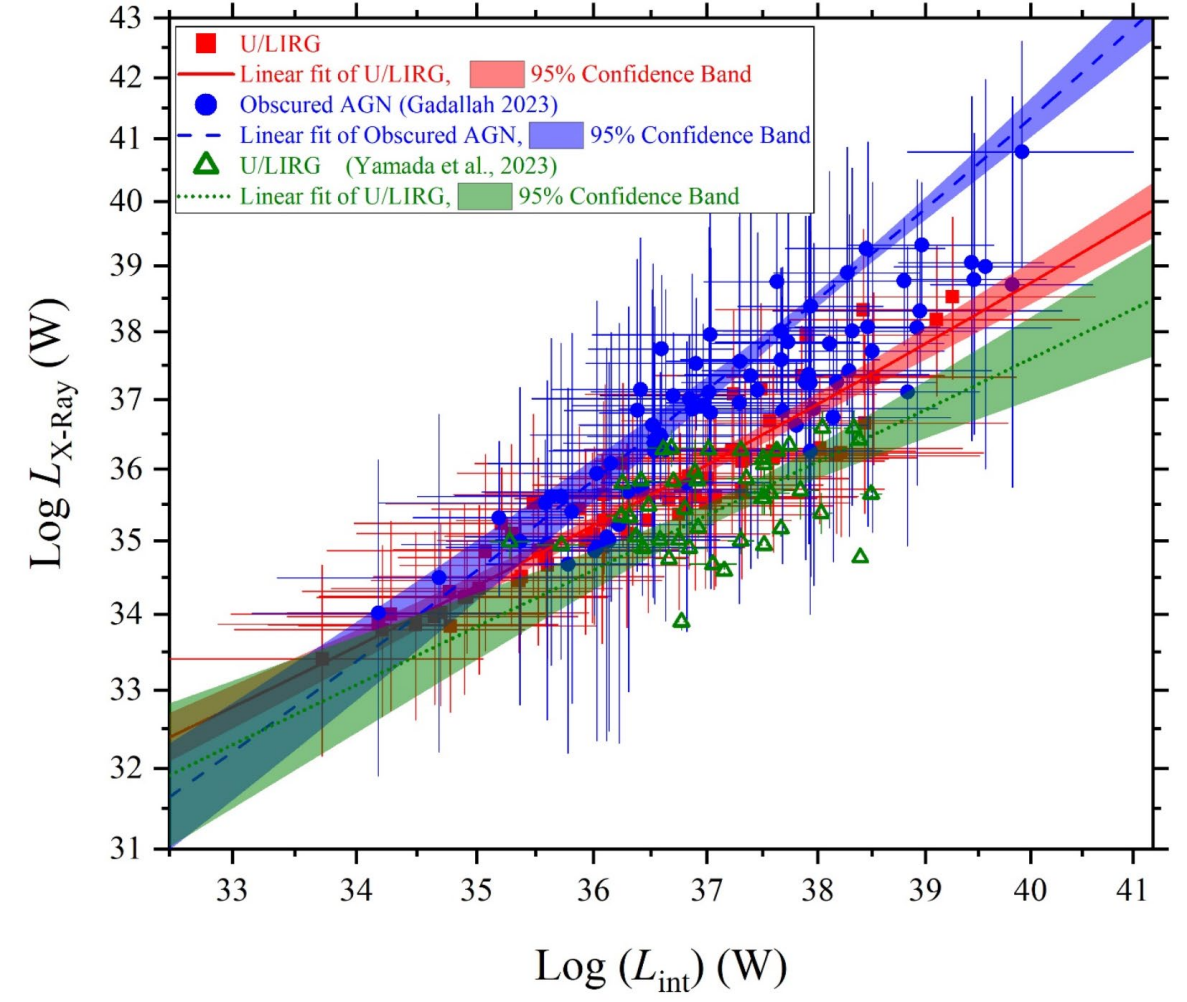
The X-ray luminosity versus the intrinsic luminosity of U/LIRGs compared with those of other U/LIRGs^41^ and obscured AGN galaxies^24^.

As we are concern in characterizing the SED luminosity components of the U/LIRG galaxies, we compare their variations versus the intrinsic luminosity with those of the obscured AGN galaxies which is similar in highly emitted IR emissions due to the existence of the dusty torus. Both U/LIRG and obscured AGN galaxies are similarly fueled by an energetically active nucleus which is surrounded by a dusty torus. The decomposed SED luminosity components can be used to differentiate between them, Figs. 15, 16 and 17 compare the variation of the luminosity components of U/LIRGs to those of obscured AGN galaxies clarified by^24^ in relative to the intrinsic AGN power. In this comparison, it is clear that the variation of both the stellar and X-ray luminosities with increasing the intrinsic power of the nucleus is somehow faster of obscured AGN than those of U/LIRG, showing that this luminosity is highest of obscured AGN at high values of the intrinsic power. But for the variation of the AGN luminosity of both, they have a similar trend variation. For X-ray luminosity of our sample of U/LIRG compared to other similar sample of U/LIRG by^41^ as presented in Fig. 17, there is a god agreement in their variation with the intrinsic luminosity.

**Fig. 18:**
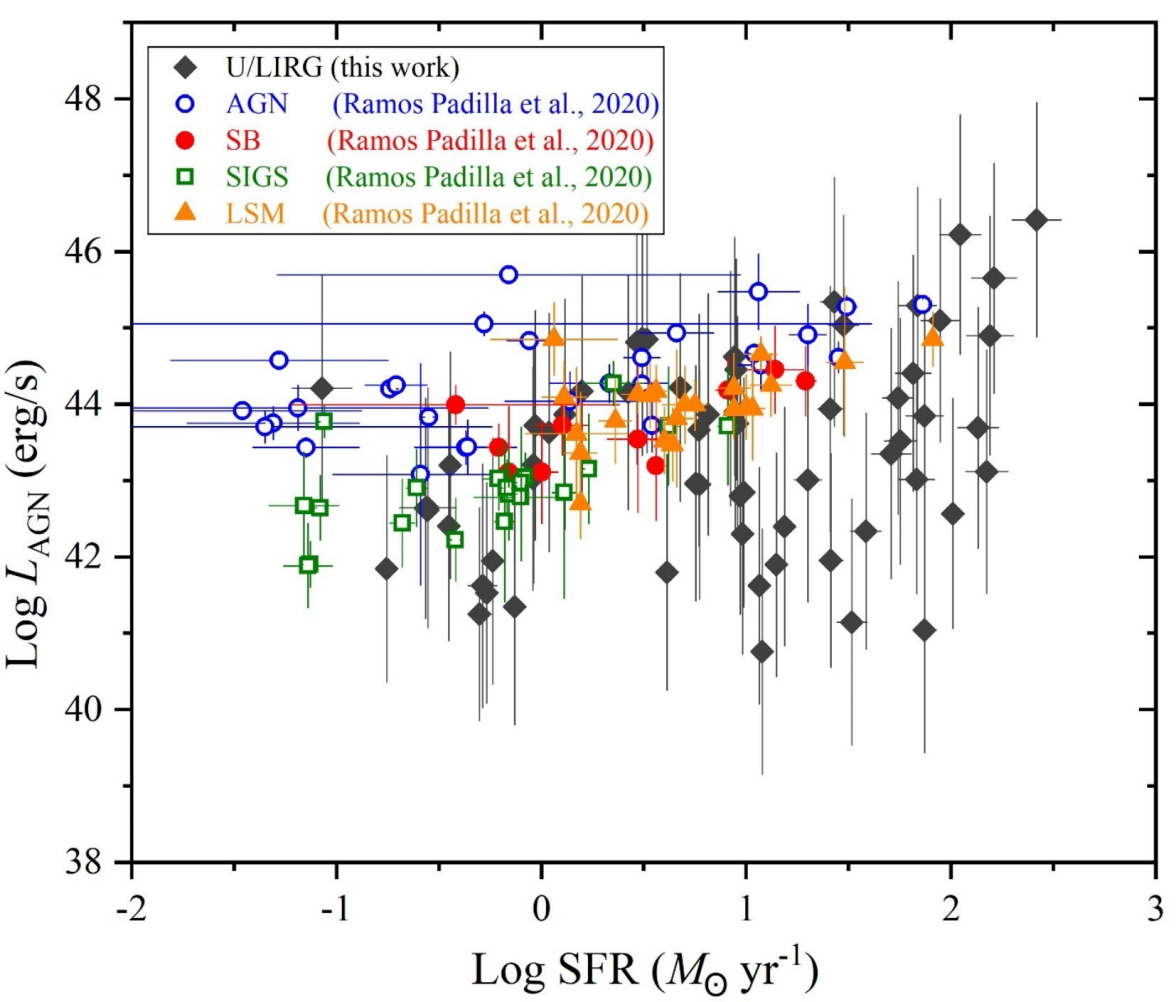
The AGN luminosity of different type of sources versus the SFR. Values of AGN (open circles), SB (closed circles), SIGS (open squares), and LSM (closed trianles) are taken from^92^.

**Fig. 19:**
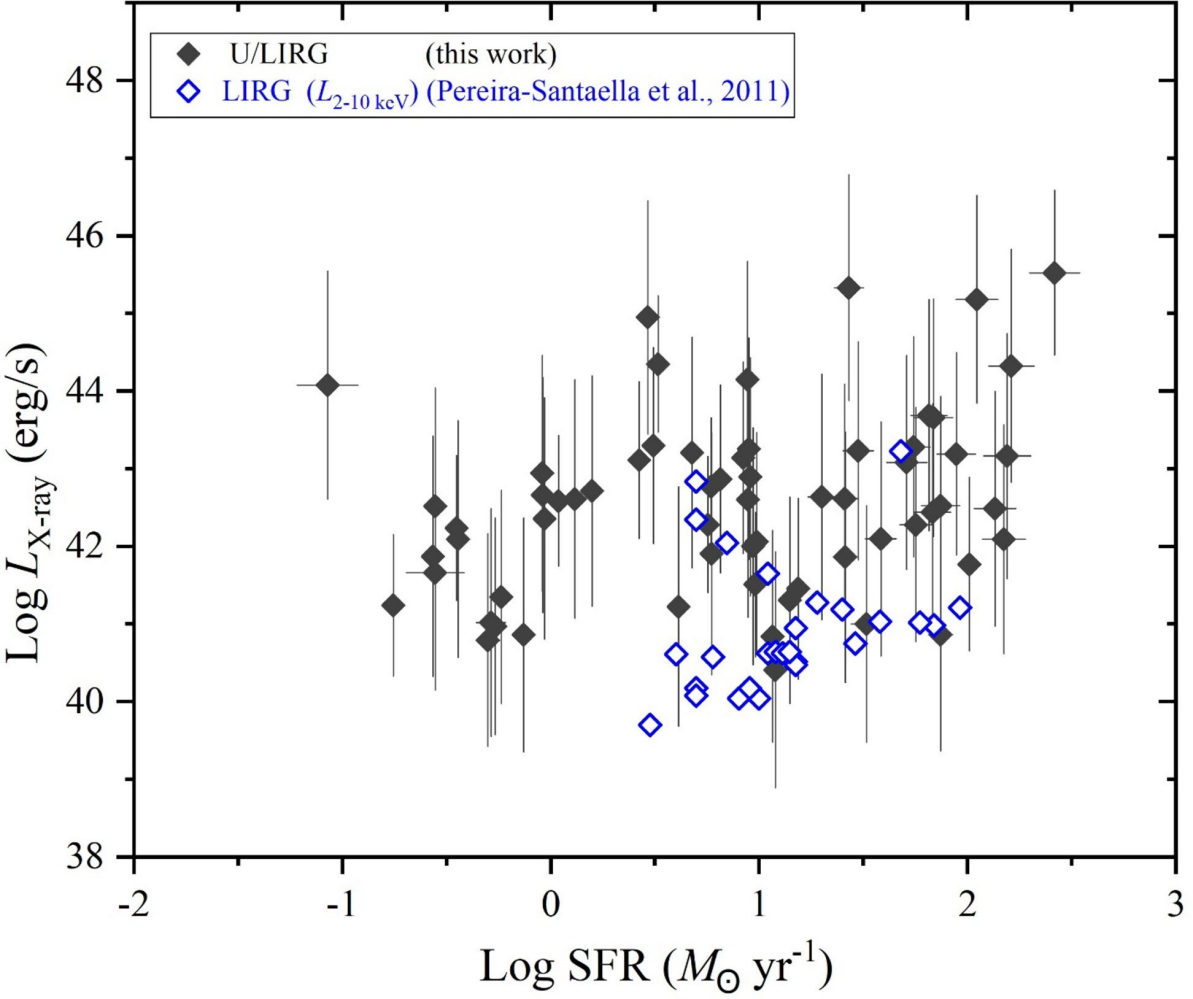
The X-ray luminosity of U/LIRGs compared with other LIRG (open symbols) sources^93^ versus the SFR.

For the decomposed AGN and X-ray luminosities in erg s^−1^, Figs. 18 and 19, respectively, compare them with those from literatures for similar and different galaxies with variation of the SFR. Figure 18 presents the AGN luminosity of our sample compared with those for various types of galaxies^92^. These galaxies include AGN galaxies characterized by their strong neon emission lines of high ionizing flux, Starburst galaxies (SB) dominated by star formation, the Spitzer Interacting Galaxies Sample (SIGS) being relatively bright and identified as spiral galaxies with a companion seen in close projection, and Late-Stage Merging (LSM) galaxies being in or approaching their final coalescence. In this comparison, it is shown that all samples of^92^ have similar ranges of the AGN luminosity agreed with those of U/LIRG. Compared to the SFR of U/LIRG, SB, SIGS, and LSM have noticeable different ranges of their SFR. SIGS sample agrees with U/LIRGs at low SFR while both SB and LSM appear at high SFR. For AGN sample, their SFR is almost like that of U/LIRG. Versus the SFR, Fig. 19 presents the X-ray luminosity of U/LIRGs compared to those of similar sample of LIRGs in the energy band 2–10 keV^93^. It is shown that the 2–10 keV luminosity of LIRG appear slightly lower than that of U/LIRG but LIRG is characterized by its high SFR in agreement with high SFR values of U/LIRG.

**Fig. 20:**
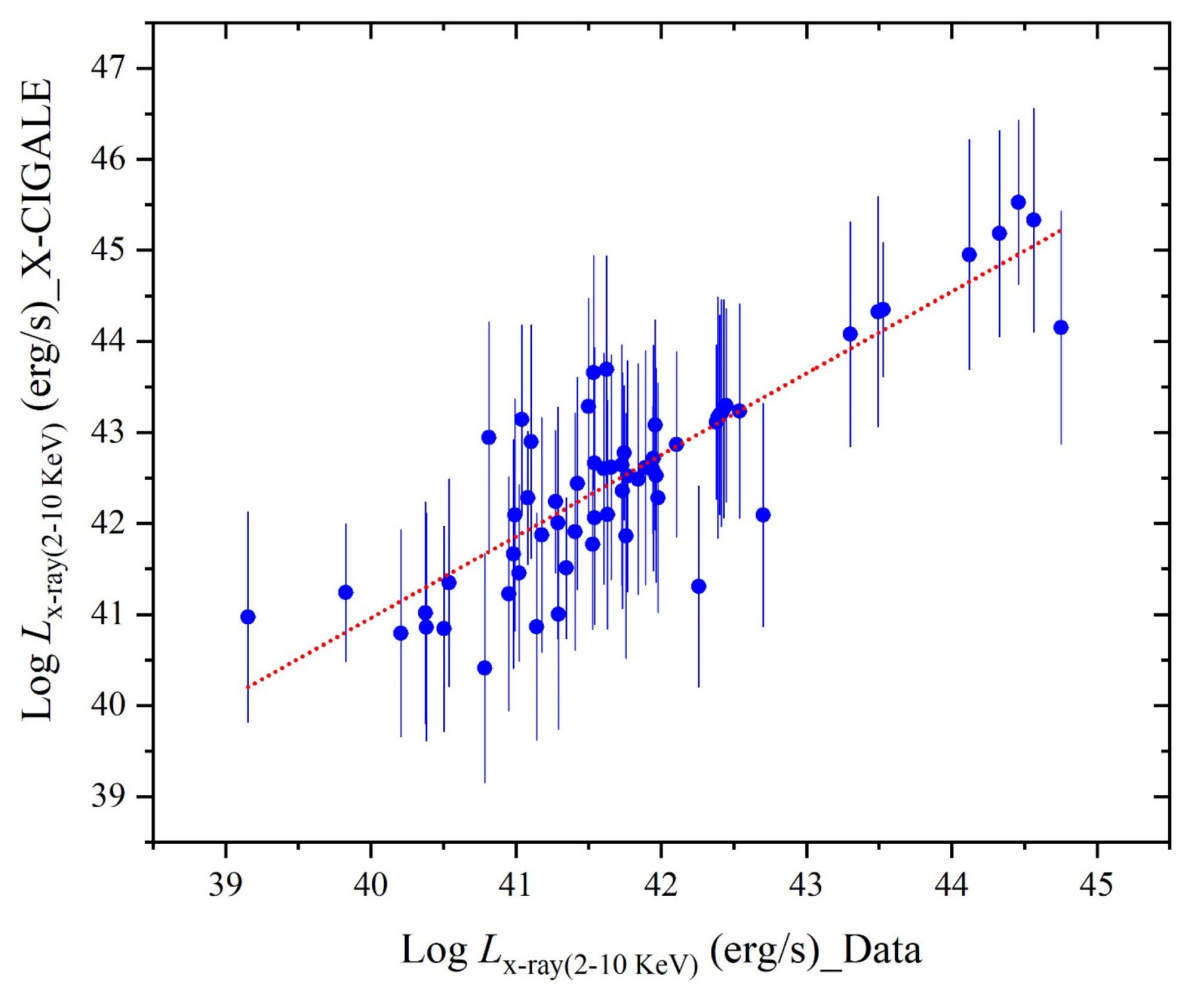
Comparison of the X-ray luminosity in the 2–10 keV band estimated by the SED fitting as an output of X-CIGALE code with those of the observed values of the sample as input data of this code, where the dotted line shows a linear fit.

As the X-ray luminosity, calculated in erg s^−1^ in the band energy of a range of 2–10 keV, is one of the outputs of X-CIGALE code, Fig. 20 presents it in comparison with that of adapted input data (observations) shown in Fig. 1, in the same band energy. This comparison shows a good agreement between calculated and observed values of this luminosity of the U/LIRGs where a tight linear fit is found with a slope of 0.896 ± 0.065.

## Conclusion

For U/LIRG galaxies, SED fittings spanning a wide range of wavelengths from X-ray to FIR can produce many important physical properties. From which, both the stellar, gas and dust masses and SFR are considered in addition to the luminosity and its components. Both total luminosity and its components (stellar, AGN, and X-ray luminosities) are characterized versus the galaxy mass. Generally, it is obvious that the stellar luminosity has the dominate contributions relative to the total luminosity. For this total luminosity, it has a strong correlation versus the galaxy mass while the intrinsic luminosity has an intermediate one. Regarding the luminosity components (stellar, AGN and X-ray), they have strong correlations versus both of stellar and gas masses. On the other hand, the variations of these components versus dust mass have weak correlations.

In luminosity-luminosity dependence, the variation of stellar, AGN, and X-ray luminosities with increasing the intrinsic AGN luminosity, shows upward trends but with various rates of variation. For which, these luminosities of U/LIRGs shows a strong correlation with the intrinsic AGN luminosity where both of AGN and X-ray have strong correlations than that of Stellar luminosity. This agrees with that of obscured AGN. Finally, relationships between different luminosity components are depicted, revealing strong correlations of stellar and X-ray luminosities with the AGN luminosity. On the other hand, the X-ray luminosity strongly corelates to the stellar luminosity but it has an intermediate variation with increasing IR luminosities. In summary, this analysis can offer valuable insights into the physical properties and their relationships of U/LIRGs.

## Electronic supplementary material

Below is the link to the electronic supplementary material.

Supplementary Material 1

## Data Availability

The datasets used and/or analyzed during this study available from the corresponding author on reasonable request.
