# Supplementary material for: Characterizing the luminosity components of luminous infrared galaxies in multi-wavelength from the X-ray to the far-infrared
Source: Sci Rep. 2024 Oct 27;14:25648. doi: 10.1038/s41598-024-76203-5 (PMC11514313; doi:10.1038/s41598-024-76203-5)
Supplement: Supplementary file 1 — Supplementary Material 1 [file 41598_2024_76203_MOESM1_ESM.docx]

**Appendix A**

Table A1. Galaxy sample of U/LIRG galaxies.

| Sample | *z* | *Merger type* | *i*° | *oa*° | *R*_out_/*R*_in_ | Log *L*_IR_ (*L*_☉_) | SFR  (*M*_☉_yr^-1^) | *L*_X-ray_  (10^42^ erg s^-1^) | *f*_AGN_ | *χ*^2^ |
| --- | --- | --- | --- | --- | --- | --- | --- | --- | --- | --- |
| IRASF10214+4724 | 2.2856 | x | 30 | 50 | 20 | 13.45^d^ | 260.95 | 3362.04 | 0.1 | 2.3 |
| SDSS_J120226.76-012915.2 | 0.1504 | x | 30 | 50 | 30 | 12.43^b^ | 161.56 | 211.66 | 0.3 | 2.9 |
| SDSS_J125400.80+101112.4 | 0.31896 | x | 20 | 50 | 20 | 12.58^c^ | 154.37 | 14.61 | 0.02 | 4.2 |
| IRASF12540+5708 | 0.04217 | D (late) | 30 | 40 | 10 | 12.57^a^ | 149.03 | 1.23 | 0.001 | 1.3 |
| IRAS13120-5453 | 0.03076 | D (late) | 70 | 30 | 30 | 12.32^a^ | 135.05 | 3.07 | 0.008 | 3.4 |
| SDSS_J015950.25+002340.8 | 0.16311 | x | 20 | 30 | 30 | 12.52^b^ | 110.87 | 1524.84 | 0.8 | 1.1 |
| IRASF10565+2448 | 0.0431 | D (late) | 90 | 80 | 20 | 12.08^a^ | 102.03 | 0.59 | 0.001 | 3.5 |
| SDSS_J132419.89+053704.7 | 0.20281 | x | 90 | 50 | 10 | 12.63^d^ | 88.09 | 15.61 | 0.06 | 3.3 |
| IRASF23128-5919 | 0.0446 | C (late) | 90 | 50 | 20 | 12.06^a^ | 74.07 | 3.36 | 0.02 | 3.7 |
| IRASF01364-1042 | 0.04823 | D (late) | 90 | 50 | 20 | 11.85^a^ | 74.07 | 0.07 | 0.001 | 3.7 |
| IRASF17207-0014 | 0.04281 | D (late) | 90 | 30 | 10 | 12.46^a^ | 68.57 | 45.59 | 0.3 | 2.6 |
| IRASF14348-1447 | 0.08231 | D (late) | 30 | 30 | 10 | 12.39^a^ | 67.97 | 2.74 | 0.001 | 1.8 |
| IRASF14378-3651 | 0.06764 | D (late) | 80 | 50 | 30 | 12.23^a^ | 65.27 | 49.11 | 0.08 | 2.8 |
| IRASF20551-4250 | 0.043 | D (late) | 90 | 80 | 30 | 12.06^a^ | 56.47 | 1.90 | 0.01 | 4.3 |
| IRASF04315-0840 | 0.01594 | D (late) | 90 | 50 | 20 | 11.65^a^ | 55.06 | 19.20 | 0.08 | 1.8 |
| IRASF12112+0305 | 0.07332 | D (late) | 30 | 30 | 30 | 12.36^a^ | 51.02 | 12.11 | 0.002 | 2.6 |
| IRASF09111-1007 | 0.05414 | B (early) | 90 | 50 | 30 | 12.06^a^ | 38.34 | 1.25 | 0.001 | 4 |
| IRASF01173+1405 | 0.03152 | B (early) | 90 | 30 | 30 | 11.69^a^ | 32.72 | 0.10 | 0.001 | 2.9 |
| IRASF13428+5608 | 0.03734 | D (late) | 90 | 50 | 30 | 12.21^a^ | 29.87 | 17.07 | 0.3 | 2.1 |
| SDSS_J171433.73+592119.3 | 0.906 | x | 80 | 10 | 10 | 11.7^e^ | 26.93 | 2152.69 | 0.5 | 1.1 |
| IRASF11095-0238 | 0.10663 | x | 80 | 50 | 10 | 12.2^c^ | 25.88 | 0.73 | 0.003 | 2.2 |
| IRASF13136+6223 | 0.03079 | B (early) | 90 | 30 | 30 | 11.81^a^ | 25.71 | 4.13 | 0.05 | 3.2 |
| IRASF00456-2904 | 0.10989 | x | 90 | 50 | 10 | 12.12^c^ | 20.02 | 4.37 | 0.001 | 4.5 |
| IRASF18093-5744 | 0.01734 | B (early) | 90 | 80 | 30 | 11.62^a^ | 15.40 | 0.28 | 0.002 | 2.4 |
| IRASF03316-3618 | 0.00546 | N | 30 | 50 | 10 | 11^a^ | 14.024 | 0.20 | 0.001 | 1 |
| IRASF16399-0937 | 0.02701 | D (late) | 90 | 30 | 30 | 11.63^a^ | 11.96 | 0.02 | 0.001 | 4.2 |
| IRASF23133-4251 | 0.00536 | N | 90 | 50 | 30 | 11.11^a^ | 11.59 | 0.06 | 0.001 | 1.8 |
| IRASF03117+4151 | 0.02334 | N | 90 | 50 | 10 | 11.41^a^ | 9.72 | 1.15 | 0.007 | 2.7 |
| IRASF11257+5850 | 0.01041 | C (late) | 90 | 50 | 10 | 11.93^a^ | 9.58 | 0.32 | 0.001 | 2.4 |
| IRASF13229-2934 | 0.01369 | N | 90 | 30 | 20 | 11.3^a^ | 9.32 | 1.01 | 0.01 | 3.1 |
| IRASF20304-0211 | 0.02001 | D (late) | 90 | 50 | 30 | 11.32^a^ | 9.05 | 7.93 | 0.09 | 1.9 |
| IRASF23254+0830 | 0.02903 | A | 70 | 50 | 10 | 11.56^a^ | 8.92 | 18.10 | 0.2 | 1.7 |
| IRASF00085-1223 | 0.01962 | D (late) | 90 | 50 | 30 | 11.49^a^ | 8.83 | 4.01 | 0.07 | 2.7 |
| IRASF03164+4119 | 0.01756 | N | 30 | 30 | 30 | 11.2^a^ | 8.76 | 141.83 | 0.5 | 3.4 |
| IRASF21453-3511 | 0.01615 | N | 90 | 30 | 30 | 11.42^a^ | 8.40 | 13.93 | 0.2 | 3.9 |
| IRASF13362+4831 | 0.02786 | B | 80 | 30 | 30 | 11.56^a^ | 6.51 | 7.38 | 0.06 | 1.6 |
| IRASF00344-3349 | 0.0206 | N | 90 | 30 | 30 | 11.28^a^ | 5.917 | 0.812 | 0.02 | 2.6 |
| SDSS_J090634.03+045127.6 | 0.12498 | x | 90 | 50 | 10 | 12.07^d^ | 5.88 | 5.93 | 0.04 | 0.3 |
| IRASF04118-3207 | 0.01191 | N | 70 | 50 | 10 | 11.07^a^ | 5.674 | 1.91 | 0.001 | 0.3 |
| IRASF13197-1627 | 0.01654 | D (late) | 80 | 60 | 30 | 11.28^a^ | 4.763 | 0.11 | 0.35 | 5.6 |
| IRASF15250+3608 | 0.05521 | D (late) | 90 | 80 | 30 | 12.08^a^ | 4.10 | 0.168 | 0.001 | 6.4 |
| IRASF23007+0836 | 0.01627 | A | 60 | 20 | 20 | 11.65^a^ | 3.27 | 224.36 | 0.35 | 2.9 |
| IRASF16504+0228 | 0.02431 | D (late) | 90 | 50 | 30 | 11.93^a^ | 3.11 | 19.84 | 0.35 | 3.3 |
| SDSS_J134733.36+121724.3 | 0.12 | x | 50 | 80 | 20 | 12.28^d^ | 2.91 | 896.08 | 0.35 | 4.8 |
| IRAS20264+2533 | 0.0139 | D (late) | 90 | 40 | 20 | 11.11^a^ | 2.65 | 13.01 | 0.35 | 3.6 |
| IRAS23262+0314 | 0.01715 | B (early) | 70 | 50 | 10 | 11.11^a^ | 1.57 | 5.20 | 0.32 | 2.3 |
| IRASF23365+3604 | 0.06448 | D (late) | 60 | 80 | 20 | 12.2^a^ | 1.29 | 4.09 | 0.2 | 3.9 |
| IRASF05189-2524 | 0.04275 | D (late) | 70 | 50 | 10 | 12.16^a^ | 1.08 | 3.86 | 0.008 | 1.6 |
| IRASF08572+3915 | 0.0582 | D (late) | 80 | 80 | 30 | 12.16^a^ | 0.93 | 2.29 | 0.01 | 4.1 |
| IRASF23135+2517 | 0.02742 | N | 90 | 30 | 10 | 11.6^a^ | 0.91 | 4.59 | 0.02 | 1.8 |
| IRASF11231+1456 | 0.03401 | A (early) | 90 | 50 | 10 | 11.64^a^ | 0.90 | 8.76 | 0.007 | 1.8 |
| IRASF01417+1651 | 0.02744 | A (early) | 90 | 30 | 30 | 11.64^a^ | 0.73 | 0.07 | 0.001 | 4.8 |
| IRASF05365+6921 | 0.01312 | B (early) | 90 | 50 | 10 | 11.06^a^ | 0.57 | 0.22 | 0.002 | 2.7 |
| IRASF12243-0036 | 0.00709 | N (early) | 90 | 30 | 10 | 11.19^a^ | 0.54 | 0.09 | 0.009 | 3.1 |
| IRASF23157+0618 | 0.01654 | N | 90 | 50 | 10 | 11.12^a^ | 0.51 | 16.26 | 0.001 | 2.8 |
| IRASF02071-1023 | 0.01284 | A (early) | 90 | 80 | 30 | 11.05^a^ | 0.49 | 0.06 | 0.008 | 3.6 |
| IRASF08354+2555 | 0.01851 | D (late) | 90 | 50 | 30 | 11.6^a^ | 0.36 | 1.24 | 0.06 | 3.9 |
| IRASF15163+4255 | 0.04019 | B (early) | 30 | 40 | 30 | 11.92^a^ | 0.35 | 1.73 | 0.001 | 2 |
| IRASF16164-0746 | 0.02715 | D (late) | 90 | 80 | 30 | 11.62^a^ | 0.28 | 0.46 | 0.004 | 2.8 |
| IRASF15327+2340 | 0.0184 | D (late) | 20 | 30 | 10 | 12.28^a^ | 0.27 | 0.74 | 0.005 | 3.4 |
| IRASF13126+2453 | 0.01291 | N | 90 | 50 | 10 | 11.14^a^ | 0.18 | 0.17 | 0.01 | 2.7 |
| IRASF09320+6134 | 0.03937 | D (late) | 20 | 30 | 10 | 12.01^a^ | 0.17 | 3.31 | 0.001 | 1.4 |
| IRASF14544-4255 | 0.01573 | A (early) | 90 | 80 | 30 | 11.23^a^ | 0.09 | 119.99 | 0.3 | 3.4 |

*Note*. The values of Log *L*_IR_ were taken from ^59^(a); ^94^(b); ^76^(c); ^9^(d); ^95^(e). Merger phase determined using high-spatial-resolution images (e.g., ^63^)where A, B, C, and D classes are mergers while N and x are nonmerger and unknown, respectively. Early (A and B) and late (C and D) mergers as stated in ^96^. The columns of *i*°, *oa*°, and *R*_out_/*R*_in_ represent the geometrical parameters including of the inclination, opening angle, and the inner to outer radius ratio, respectively. The values of these columns and those of SFR and *L*_X-ray_ (in erg s^-1^) are as estimated by the output file "bayes" values of X-CIGALE while those of the AGN fraction (*f*_AGN_) and the reduced chi-square (*χ*^2^) are taken from "best" values.

Table A2. The best-fitting parameters of the galaxy main sequence of the SFR-*M*_st_ relation (equation 5 in ^80^).

| Redshift range | S_0_ | M_0_ | *α* | *β* |
| --- | --- | --- | --- | --- |
| 0.005 < *z* < 0.3 | 1.83618 | 11.55 | 0.8 | 0.05 |
| 0.3 < *z* < 2.03 | 2.4 | 12.1 | 0.92 | 0.01 |
